# lncRNA ZNRD1-AS1 promotes malignant lung cell proliferation, migration, and angiogenesis via the miR-942/TNS1 axis and is positively regulated by the m^6^A reader YTHDC2

**DOI:** 10.1186/s12943-022-01705-7

**Published:** 2022-12-30

**Authors:** Jin Wang, Lirong Tan, Xueting Yu, Xiyuan Cao, Beibei Jia, Rui Chen, Jianxiang Li

**Affiliations:** 1 School of Public Health, Suzhou Medical College of Soochow University, Suzhou, 215123 Jiangsu China; 2 Department of Respiratory Medicine, The Second Affiliated Hospital of Soochow University, Suzhou Jiangsu, 215004 China

**Keywords:** lncRNA ZNRD1-AS1, Cigarette smoke, Lung cancer, m^6^A RNA methylation, Immune infiltration, miR-942, TNS1

## Abstract

**Rationale:**

Lung cancer is the most prevalent form of cancer and has a high mortality rate, making it a global public health concern. The N^6^-methyladenosine (m^6^A) modification is a highly dynamic and reversible process that is involved in a variety of essential biological processes. Using in vitro, in vivo, and multi-omics bioinformatics, the present study aims to determine the function and regulatory mechanisms of the long non-coding (lnc)RNA zinc ribbon domain-containing 1-antisense 1 (ZNRD1-AS1).

**Methods:**

The RNAs that were bound to the m^6^A ‘reader’ were identified using YTH domain-containing 2 (YTHDC2) RNA immunoprecipitation (RIP)-sequencing. Utilizing methylated RIP PCR/quantitative PCR, pull-down, and RNA stability assays, m^6^A modification and ZNRD1-AS1 regulation were analyzed. Using bioinformatics, the expression levels and clinical significance of ZNRD1-AS1 in lung cancer were evaluated. Using fluorescent in situ hybridization and quantitative PCR assays, the subcellular location of ZNRD1-AS1 was determined. Using cell migration, proliferation, and angiogenesis assays, the biological function of ZNRD1-AS1 in lung cancer was determined. In addition, the tumor suppressor effect of ZNRD1-AS1 in vivo was validated using a xenograft animal model. Through bioinformatics analysis and in vitro assays, the downstream microRNAs (miRs) and competing endogenous RNAs were also predicted and validated.

**Results:**

This study provided evidence that m^6^A modification mediates YTHDC2-mediated downregulation of ZNRD1-AS1 in lung cancer and cigarette smoke-exposed cells. Low levels of ZNRD1-AS1 expression were linked to adverse clinicopathological characteristics, immune infiltration, and prognosis. ZNRD1-AS1 overexpression was shown to suppress lung cancer cell proliferation, migration, and angiogenesis in vitro and in vivo*,* and to reduce tumor growth in nude mice. ZNRD1-AS1 expression was shown to be controlled by treatment of cells with either the methylation inhibitor 3-Deazaadenosine or the demethylation inhibitor Meclofenamic. Furthermore, the miR-942/tensin 1 (TNS1) axis was demonstrated to be the downstream regulatory signaling pathway of ZNRD1-AS1.

**Conclusions:**

ZNRD1-AS1 serves an important function and has clinical relevance in lung cancer. In addition, the findings suggested that m^6^A modification could mediate the regulation of the ZNRD1-AS1/miR-942/TNS1 axis via the m^6^A reader YTHDC2.

**Graphical Abstract:**

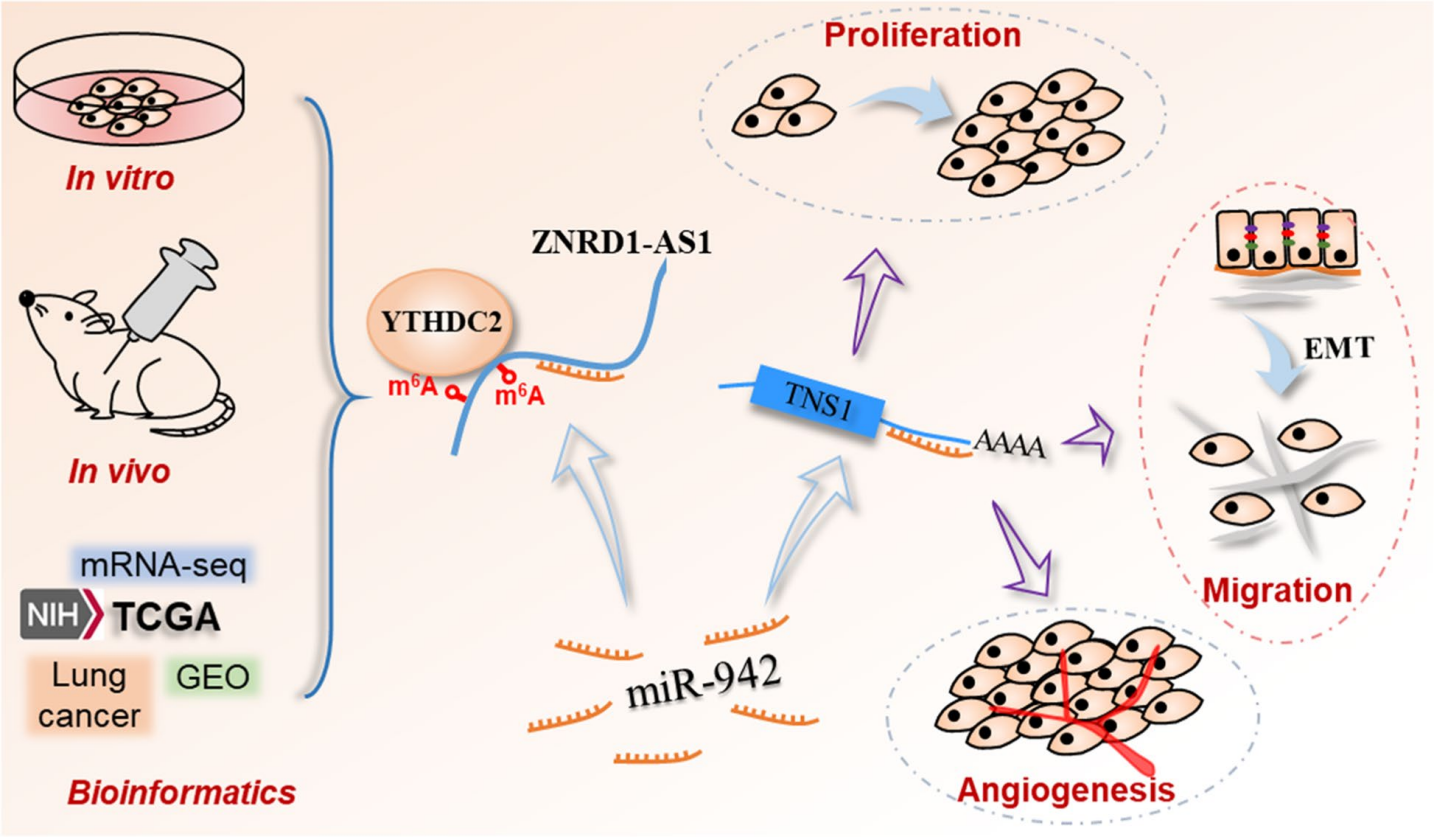

**Supplementary Information:**

The online version contains supplementary material available at 10.1186/s12943-022-01705-7.

## Introduction

Lung cancer has the highest cancer mortality rate and is a significant public health problem worldwide [1]. Cigarette smoking is the primary cause of lung cancer and epidemiological studies have also revealed that the risk of lung cancer is associated with the frequency and duration of smoking over an individual’s lifetime [2]. A recent study, using single-cell somatic mutation analysis, reported that smoking increases the risk of lung cancer by elevating the proportion of somatic mutations [3]. However, there are still numerous patients with lung cancer who do not possess these genetic mutations, or whereby treatment has little effect in patients with these mutated genes, the mechanism of which is unknown.

With the development of high-throughput sequencing and analysis technologies, it has been reported that < 2% of all transcripts encode proteins, while most transcripts are non-coding RNAs (ncRNAs) [4]. Long non-coding RNAs (lncRNAs) are a class of ncRNAs > 200 nucleotides. An increasing number of studies have demonstrated that lncRNAs serve important roles in various key biological processes, including cell proliferation, apoptosis, migration and invasion [5–7]. An increasing number of dysregulated lncRNAs have been reported to regulate biological functions in lung cancer and are regarded as potential biomarkers for lung cancer diagnosis or as therapeutic targets [8, 9]. For example, the lncRNA HOX transcript antisense RNA is able to repress gene expression by recruiting chromatin modifiers and can promote lung cancer cell proliferation, migration, invasion and drug resistance [9]. Moreover, zinc ribbon domain-containing 1 (ZNRD1)-antisense 1 (AS1) encodes a protein that potentially contributes to the progression of cancer and HIV infection [10, 11]. Previous studies have demonstrated that ZNRD1-AS1 is upregulated and is described as an oncogene in several types of cancer, including liver, cervical and endometrial cancers [11–13].

N^6^-methyladenosine (m^6^A) is the most common widespread modification on various types of RNA. m^6^A on transcripts can affect a range of different cellular biological processes, including the splicing, nuclear transport, stabilization and translation of RNAs [14, 15]. Furthermore, it has been reported that m^6^A modification is also present in numerous ncRNAs [16–18]. Previous studies of this type of modification have demonstrated that m^6^A serves important functions in diverse biological processes and disease progression, including in early development, viral infection and cancer [19–22]. m^6^A methylation and demethylation are performed by a methylase ‘writer’ and demethylase ‘eraser’, respectively. However, what determines the m^6^A modification fate is the ‘reader’ protein, which can recognize and interact with m^6^A containing RNA to regulate its fate and functions. A recent study demonstrated that exposure to cigarette smoke (CS) extract can induce the upregulated expression of the m^6^A methylase methyltransferase 3 (METTL3), and the increased m^6^A modification of the 3’untranslated region (UTR) of the transcriptional repressor zinc finger and BTB domain-containing 4 in human bronchial epithelial cells [23]. Moreover, previous studies have reported that m^6^A methylation can affect numerous properties of tumor cells via the regulation of lncRNAs. For example, in liver cancer, enhancing LINC00958 stability via METTL3 m^6^A modification results to its upregulation, which thereby promotes cancer progression [24]. Furthermore, the demethylase AlkB homolog 5 (ALKBH5) can regulate the stability of potassium two-pore domain channel subfamily K member 15 (KCNK15)-AS1 via demethylation and inhibit the migration and invasion of pancreatic cancer cells [25]. In our previous study it was demonstrated that YTH domain-containing 2 (YTHDC2), an m^6^A ‘reader’, was reduced in lung cancer and contributed to cell proliferation, migration and the epithelial-mesenchymal transition (EMT) process. Moreover, YTHDC2 may serve as a tumor suppressor via the m^6^A-mediated CYLD lysine 63 deubiquitinase/NF-κB regulatory signaling pathway [26].

The present study aimed to explore the expression profile, biological functions and m^6^A-mediated regulatory mechanism of lncRNA ZNRD1-AS1 via the m^6^A reader YTHDC2 by in vitro and in vivo studies, as well as bioinformatic analysis.

## Materials and methods

### *In vitro* cell model of CS-induced malignant transformation

The normal human bronchial epithelial BEAS-2B cell line (cat. no. CRL-9609) was purchased from the American Type Culture Collection and was used to establish an in vitro model of CS-induced malignant transformation, as previously described [27]. Briefly, aliquots of exponentially growing 1 × 10^5^ BEAS-2B cells were plated onto a Transwell membrane (0.4-μm pore, Corning, Inc.). An automatic smoking machine (cat. no. MED8170A, Tianjin Hope Industry & Trade Co., Ltd.) was used to produce CS, which was then pumped into an inhalation chamber where the BEAS-2B cells were directly exposed to CS for 10 min every other day at a smoke concentration of 20%. This procedure was performed to expose the cells to CS for 10, 20 and 30 passages, and such cells were referred to as experimental S10, S20 and S30 cells, respectively. The unexposed BEAS-2B cells were used as control cells.

### Pull-down assay

Primers for ZNRD1-AS1 segmental amplification were designed. Primer sequences are presented in Table S1. Biotin-labeled RNA was synthesized via in vitro transcription using a RiboTM RNAmax-T7 biotin-labeled transcription kit (Ribobio, China). Streptavidin-labeled magnetic beads were then incubated with biotin-labeled RNA. Cells were lysed using cell lysate pre-supplemented with protease inhibitors. All molecules capable of binding to RNA were then eluted via incubation with cell lysate using magnetic beads bound to RNA. Proteins in the eluate were further detected via western blotting.

### Plasmid construction and transfection

The full-length of ZNRD1-AS1 (Ensembl ID: ENST00000431012) was amplified using Pfu DNA Polymerase (Sangon Biotech Co., Ltd., China) and ligated into the pCDH overexpression vector (pZNRD1-AS1). ZNRD1-AS1 small hairpin (sh)RNAs were designed using the BLOCK-iT™ RNAi Designer. Subsequently, the shRNA pairs were annealed and ligated into the pGreen vector (shZNRD1-AS1). The H1299 and S30 cells were transfected with pZNRD1-AS1 and shZNRD1-AS1 using Lipofectamine® 6000 (Beyotime Institute of Biotechnology, China) according to the manufacturer’s protocol.

### RT-qPCR

After centrifugation of the cells, 1 ml TRIzol (Invitrogen, Thermo Fisher Scientific, Inc., Waltham, MA, USA) was added per 5 × 10^6^ cells. Subsequently, total RNA was extracted according to the manufacturer’s protocol. Total RNA (~ 1.5 μg) was reverse transcribed into complementary DNA (cDNA) applying a RevertAid First Strand cDNA Synthesis Kit (Thermo Fisher Scientific, Inc., Waltham, MA, USA) according to the manufacturer’s protocol. Subsequently, qPCR was performed using FastStart Universal SYBR Green Master (ROX) Kit (Roche Diagnostics, Switzerland) and the QuantStudioTM 6 Flex RT-qPCR System (Applied Biosystems, Thermo Fisher Scientific, Inc., Foster City, CA, USA). GAPDH was used as the internal reference gene. The qPCR primer pairs used are presented in Table S2.

### RNA stability assay

The RNA stability assay was performed as previously described [28], and the YTHDC2 overexpression plasmid was constructed in our previous study [26]. The YTHDC2 overexpression and control H1299 cells were seeded onto 6-well plates and were treated with actinomycin D (final concentration, 10 μg/ml). Cells were harvested at different time points (0, 1, 2, 3, 4 and 5 h) following the addition of actinomycin D. Total RNA was isolated using TRIzol® reagent and expression levels were assessed using quantitative (q)PCR. Ct values at different time points were normalized to the Ct value at t = 0 to obtain the ∆Ct value (∆Ct = average Ct of each time point—average Ct of t = 0), and the relative RNA abundance at each time point was calculated using the following formula: 2^−∆CT^. The mRNA decay rate was determined using a non-linear regression curve fitting (one phase decay) using GraphPad Prism V9.1 (GraphPad Software, Inc.).

### m^6^A RNA immunoprecipitation (meRIP) assay

M^6^A modification in ZNRD1-AS1 was predicted according to the sequence-based RNA adenosine methylation site predictor (SRAMP) online tool [29]. m^6^A site-specific primer pairs were designed for meRIP PCR/qPCR based on the predicted high confidence m^6^A sites. The m^6^A modification in S30 and H1299 cells was verified by using the Magna MeRIP m^6^A Kit (cat. no. 17–10,499, Millipore Sigma, MA, USA) according to the manufacturer’s instructions. In brief, 150 μg total RNA was randomly fragmented and then immunoprecipitated with anti-m^6^A antibody-coated magnetic beads. The m^6^A-contained RNA fragments were enriched and then eluted. The abundance of eluted-RNA was quantified via PCR and reverse transcription (RT)-qPCR assays. The relative enrichment of m^6^A in RNA segments was normalized to the input.

### Intervention of m^6^A modification

The level of m^6^A modification was interfered with using 3-deazaadenosine (3-DAA) and meclofenamic acid (MA). 3-DAA is a broad-spectrum global methylation inhibitor (work concentration: 50 μM). MA, a non-steroidal anti-inflammatory agent, is a highly selective inhibitor of fat mass and obesity-associated (FTO) and is able to compete with FTO for m^6^A containing nucleic acids (work concentration: 100 μM). Cells were collected for subsequent analysis after 24 h of treatment.

### Data analysis using The Cancer Genome Atlas (TCGA) and Gene Expression Omnibus (GEO) databases

The Genotype-Tissue Expression (GTEx, https://gtexportal.org/) project samples are derived from over 7,000 autopsy samples from 449 antemortem healthy human donors covering 42 different tissue types. Transcripts per million (TPM) normalized GTEx and TCGA pan-cancer data, and associated sample annotation information, were obtained using the Xena browser (https://xenabrowser.net/). The GTEx and pan-cancer data were segmented and merged via tissue type using R software (Version 4.1.1) to compensate for the deficiency of insufficient normal tissue samples in the TCGA database. Subsequently, the expression profiles of specific genes were extracted and expression quantity analysis of ZNRD1-AS1 in different cancer types was performed.

GEO (https://www.ncbi.nlm.nih.gov/geo/query/acc.cgi) is an international public repository for archiving and the free sharing of investigator submitted microarray, high-throughput sequencing and other data. By searching the GEO database, a total of four lung cancer datasets, namely GSE41271[30] and GSE30219 [31], for clinicopathological and survival analysis, and GSE5372 [32] and GSE5059 [33] for expression difference analysis, were identified.

The gene expression data were grouped according to lung cancer stage, distal metastasis, lymph node metastasis and invasion depth and the gene expression differences between the groups were analyzed.

### Survival analysis

Probes for ZNRD1-AS1 were retrieved using the sequencing platform annotation information available on the online Kaplan–Meier plotter (http://kmplot.com/analysis/) [34]. Subsequently, the correlation between the ZNRD1-AS1 expression level and overall survival of patients with lung cancer was analysed using this online tool. Default values were used for this analysis and the follow-up threshold was set at 120 months (10 years).

### Correlation analysis of immune infiltration and immune checkpoints

The Tumor IMmune Estimation Resource (TIMER, http://timer.comp-genomics.org/) is a comprehensive resource for systematically analyzing the immune infiltrates in different cancer types [35]. The TIMER2.0 database contains information to explore the abundance of different types of immune cells in the tumor microenvironment of the two lung cancer types, lung adenocarcinoma (LUAD) and lung squamous cell carcinoma (LUSC). The expression data of YTHDC2 were extracted from the two lung cancer datasets, and the correlation between YTHDC2 expression levels and immune cells infiltration were analyzed using R. In total, 60 immune checkpoint genes reported in the literature (including 24 inhibitory and 36 stimulatory genes) [36] were extracted from the TCGA dataset for YTHDC2 and immune checkpoint gene expression correlation analysis.

### Fluorescence in situ hybridization (FISH)

FISH assays were performed using the LncRNA FISH Probe Mix Kit (Guangzhou RiboBio Co., Ltd., Guangdong, China). Briefly, cells were fixed with 4% paraformaldehyde and treated with proteinase K and permeabilization solution. Following pre-hybridization at 37 °C for 30 min, cells were hybridized with fluorescently labeled ZNRD1-AS1 probe at 37℃ overnight. Nuclei were counterstained using DAPI and imaged using a confocal laser microscope.

### Western blotting

Total protein was extracted from cells by using RIPA lysing buffer (Beyotime, China) and quantitated by using an Enhanced BCA Kit (Beyorime, China). Total protein (30 µg) was separated via SDS-PAGE and transferred onto a PVDF membrane (MilliporeSigma, Billerica, MA). After blocking with 5% BSA, the membrane was incubated at 4 °C overnight with primary antibodies for E-cadherin (CDH1, 1:1,000 dilution, ProteinTech Group, Inc., USA), N-cadherin (CDH2, 1:1,000 dilution, ProteinTech Group, Inc., USA) and GAPDH (1:1,000 dilution, Cell Signaling Technology Inc., USA). Following the primary incubation the membranes were incubated with HRP-labeled secondary antibodies. The protein bands were visualized using ECL substrate and the GeneTools GBox (Syngene) system. The intensity of each band was quantified using ImageJ software (National Institutes of Health). GAPDH was used as the internal control.

### Cell proliferation assays

For cell cycle analysis, cells were trypsinized and then fixed with 70% ethanol at -20 °C overnight. The fixed cells were washed twice with PBS and incubated with 10 μg/ml RNase A and 50 μg/ml PI (Beyotime Institute of Biotechnology, China) for 30 min. The cell cycle was analyzed using BD FACS Canto II and FlowJo V10.3 software (FlowJo LLC, Ashland, OR, USA).

For the 5-Ethynyl-2’-deoxyuridine (EdU) cell proliferation assay, cells were seeded into 6-well plates with 10 μM EdU for 2 h. Following fixation with 4% paraformaldehyde and permeation with 0.3% Triton X-100 in PBS, cells were incubated with a click reaction solution (Beyotime Institute of Biotechnology, China). Images were captured within 24 h under an inverted fluorescent microscope and were analyzed using NIH ImageJ software (Version 1.8.0).

### Cell migration assays

For the wound healing assay, cells were seeded into six-well plates and allowed to grow up to 100% confluency. Then cells were cultured in fresh FBS-free medium and scratches were made with a pipette tip. Images were taken at different time points using an inverted microscope and the wound closure rate was analyzed using NIH ImageJ software (Version 1.8.0).

For the Transwell migration assay, cells were seeded in the upper chamber of the Transwell membrane (Corning, Inc., USA) with 1 ml FBS-free medium, and 2 ml complete medium was added to the lower chamber. After the cells were cultured at 37 °C for 24 h, the cells were fixed with 4% paraformaldehyde and stained with 0.5% crystal violet solution. Subsequently, the cells in the upper chamber of the Transwell membrane were wiped. Images of the migrated cells were captured under an inverted microscope and were then assessed using NIH ImageJ software (Version 1.8.0).

### HUVEC tube formation assay

The in vitro HUVEC tube-formation assay was performed as previously described [37]. The immortalized HUVECs (cat. no. CRL-1730) were obtained from the American Type Culture Collection. In brief, the conditional medium was collected from transfection cell culture flasks prior to the experiments and HUVECs in the exponential growth phase were seeded into 96 well plates pre-coated with Matrigel. The cells were then incubated under standard cell culture conditions for 4–6 h after adding the conditional medium. Cells were observed and imaged using an invert microscope. The image was quantified using the ImageJ plugin angiogenesis analyzer.

### Xenograft model

In total, 16 specific-pathogen-free grade male BALB/c-nude mice (age, 4 weeks) were housed in the barrier system of the animal center at Soochow University (Suzhou, China) and cared for in accordance with the National Institutes of Health Guide for the Care and Use of Laboratory Animals. Mice were housed and maintained in a specific pathogen-free (SPF) facility with 12 h light/dark cycles and free access to food and water. Animals did not experience undue suffering during the experiments. The protocol was approved by the Laboratory Animal Ethics Committee of the Experiment Animal Center of Soochow University (approval no. 202011A070). Cell suspensions were prepared via trypsinization and resuspended in PBS (5 × 10^7^ cells/ml), and 100 μl was injected subcutaneously into the right armpit of nude mice in the H1299 cell control group or ZNRD1-AS1 overexpression group (8 mice/group). All mice were euthanized at 21 days following implantation and the tumors were dissected. Tumor size was measured once a week, and the maximum xenograft tumor size at the endpoint less than 15 mm. Tissues were separated into two sections, whereby one was fixed with 4% paraformaldehyde for immunohistochemistry (IHC) and the other was stored at -80 °C for RT-qPCR or western blotting analysis.

### IHC

Tissue Sects. (0.3 μm) were deparaffinized and hydrated for staining. Following antigen retrieval, sections were blocked with 5% goat serum and 3% hydrogen peroxide. The sections were processed for IHC by incubating with specific primary antibodies, including CDH1 (1:100 dilution, ProteinTech Group, Inc., USA), CDH2 (1:100 dilution, ProteinTech Group, Inc., USA), ki67 (1:100 dilution, ProteinTech Group, Inc., USA), Cyclin D1 (1:100 dilution, ProteinTech Group, Inc., USA) and CD31 (1:100 dilution, Abcam, USA). Following by incubating a goat anti-rabbit secondary antibody for 20 min and subsequently with streptavidin-HRP for 30 min at room temperature. After washing, the sections were developed using DAB and were counterstained with hematoxylin and dehydrated. Average optical density was quantified using the ImageJ plugin IHC profiler.

### Target prediction

The StarBase online tool (https://starbase.sysu.edu.cn/index.php) [38] was used to predict the ceRNAs of ZNRD1-AS1 for further enrichment analysis to predict the biological function of this lncRNA.

LncBase (https://diana.e-ce.uth.gr/lncbasev3/interactions) is a database for studying lncRNA-microRNA (miR/miRNA) interactions. The predicted target miRNAs of ZNRD1-AS1 were determined after inputting the transcript number ENST00000431012 of ZNRD1-AS1. Furthermore, the miRNAs associated with ZNRD1-AS1 expression were determined based on the lung cancer dataset in TCGA database. Moreover, the target miRNAs intersected with the lncBase predicted target miRNAs, and then identified miR-942 as a target of ZNRD1-AS1.

In accordance with four miRNA target gene prediction databases, miRDB (www.mirdb.org), miRTarBase (http://mirtarbase.mbc.nctu.edu.tw/index.html), TargetScan (http://www.targetscan.org/ vert_72) and miRWalk (http://129.206.7.150/) the target genes of miR-942 were predicted. Genes associated with miR-942 expression were assessed based on the TCGA lung cancer dataset and the aforementioned prediction results intersected to obtain the final candidate target genes.

### Dual-luciferase reporter assay

The binding site of ZNRD1-AS1 and miR-942 was determined using the lncBase online tool and the binding site of miR-942 and the tensin 1 (TNS1) 3’UTR was obtained using TargetScan. Primers were designed using primer premier 5.0 software and XbaI digestion was used to amplify the binding sites and ~ 200 bp flanking sequences up- and downstream using a standard PCR program.

After 48 h of transfected with firefly luciferase plasmid pGL3 and ranilla luciferase plasmid pRL, the cells were lysed and the prepared firefly luciferase working solution was added according to the instructions (Beyotime, China), the fluorescence value was read on a fluorescence microplate reader immediately after mixing. Then the Renilla luciferase working solution was added, and then read the fluorescence value. The Renilla fluorescence was used as an internal reference to calculate the firefly fluorescence change in each group.

### Statistical analysis

GraphPad Prism V8.0 (GraphPad Software, Inc.) and R software were used for all statistical analysis. For simple statistical comparisons between two groups, when the statistical data obeyed normality and the variance was homogeneous, the Student’s t-test was used. When the statistical data obeyed normality but the variance was unequal, a corrected Student’s t-test was used. When the data did not obey the normal distribution, the nonparametric Wilcoxon rank sum test was used. One-way ANOVA was used to compare multiple groups and Tukey’s post hoc test was used for pairwise comparisons between groups if the results showed statistical differences. Repeated-measures data, including tumor volume and scratch experiments in nude mice, were tested for significance using repeated-measures ANOVA. P < 0.05 was considered to indicate a statistically significant difference.

## Results

### YTHDC2 promotes ZNRD1-AS1 stability in malignant lung cells

**Fig. 1:**
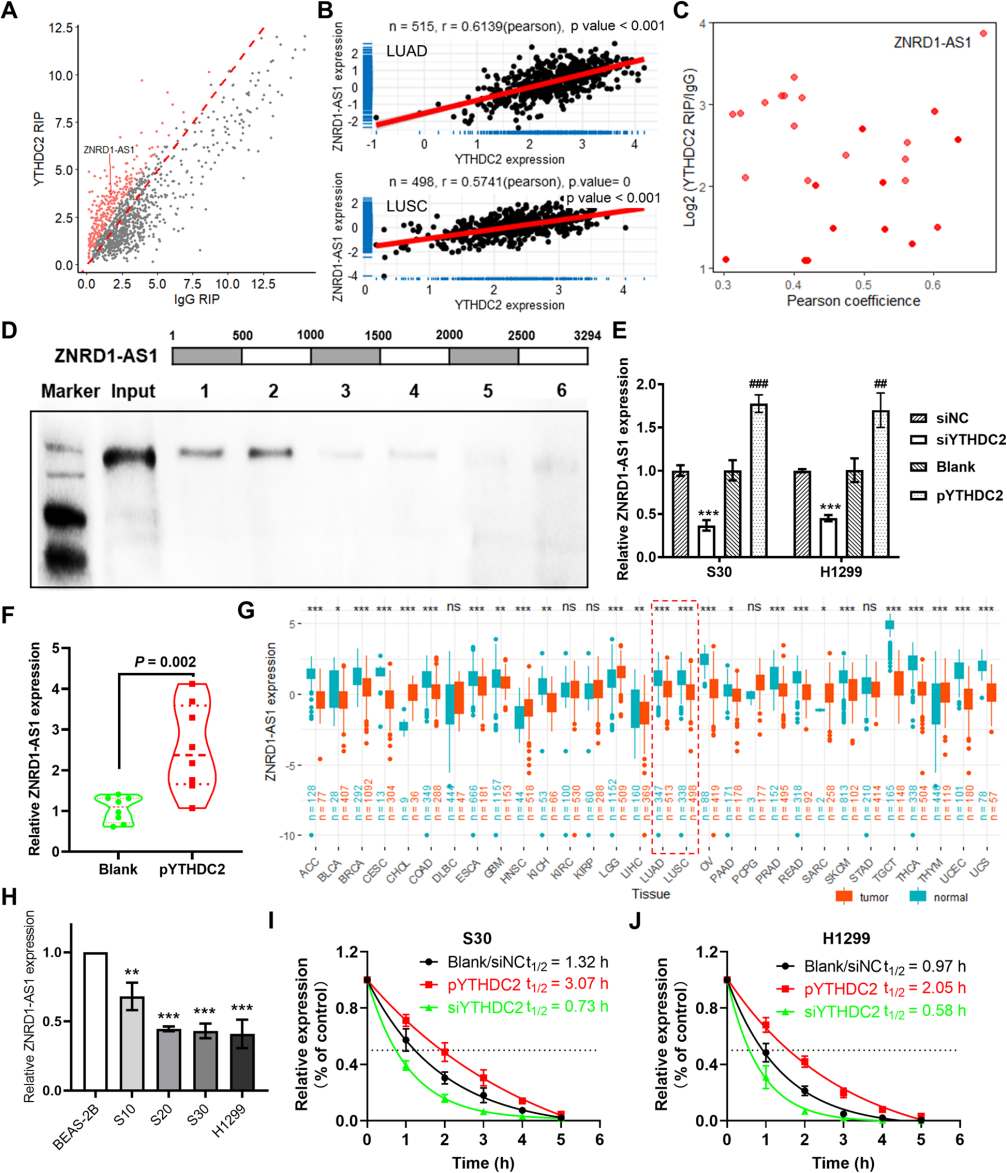
YTHDC2 promotes ZNRD1-AS1 stability in lung malignant cells. **A** YTHDC2 RIP sequencing identifies lncRNAs capable of binding to YTHDC2. **B** Correlation analysis between the expression of YTHDC2 and ZNRD1-AS1 in LUAD and LUSC datasets. **C** RIP sequencing and correlation analysis identified ZNRD1-AS1 as the putative target of YTHDC2. **D** RIP-PCR validation of YTHDC2 binding to ZNRD1-AS1. **E** qPCR was performed to detect the expression levels of ZNRD1-AS1 following the overexpression or knockdown of YTHDC2 in H1299 and S30 cells. *** *P* < 0.001 vs. siNC, ## *P* < 0.01 and ### *P* < 0.001 vs. Blank. **F** ZNRD1-AS1 expression levels in tumor formed subcutaneously by H1299 cells overexpressing YTHDC2 in nude mice. **G** Expression levels of YTHDC2 in pan-cancer based on TCGA and GTEx databases. * *P* < 0.05, ** *P* < 0.01 and *** *P* < 0.001 normal vs. tumor. **H** Relative expression of ZNRD1-AS1 in cigarette smoke-exposed BEAS-2B cells and H1299 lung cancer cells. S10, S20 and S30 represent BEAS-2B cells exposed to CS for 10, 20 and 30 passages, respectively. ** *P* < 0.01 and *** *P* < 0.01 vs. BEAS-2B group. RNA stability assay to explore the effect of overexpression or knockdown of YTHDC2 on ZNRD1-AS1 stability in (**I**) S30 and (**J**) H1299 cells. RIP: RNA immunoprecipitation. LUAD: Lung adenocarcinoma. LUSC: Lung squamous cell carcinoma. qPCR: quantitative PCR. siNC: negative control for siRNA transfection. TCGA: The Cancer Genome Atlas. GTEx: The Genotype-Tissue Expression. CS: cigarette smoke

Our previous study identified a series of lncRNAs that were potentially bound by YTHDC2 via RIP sequencing (Fig. 1A). Based on TCGA pan-cancer correlation analysis, the results demonstrated that YTHDC2 significantly and positively correlated with ZNRD1-AS1 in all tumor types, with the exception of esophageal carcinoma (R > 0.3, Figure S1A), including LUAD (R = 0.6139) and LUSC (R = 0.5741, Fig. 1B). Combined with the correlation analysis and RIP sequencing results, a total of 26 lncRNAs were identified as being significantly positively correlated with and potentially binding to YTHDC2, with ZNRD1-AS1 being the most significant (Fig. 1C). ZNRD1-AS1 was subsequently amplified in six individual segments and the proteins bound to each segment were pulled down. Western blotting demonstrated that ZNRD1-AS1 was able to bind to YTHDC2 (Fig. 1D). Furthermore, ZNRD1-AS1 expression levels were significantly increased following YTHDC2 overexpression in malignant transformed cells caused by long-term smoke exposure (S30) as well as H1299 cells; however, these expression levels were significantly decreased following ZNRD-AS1 knockdown (Fig. 1E). Moreover, ZNRD1-AS1 expression levels in tumors formed via YTHDC2-overexpressing H1299 cells implanted subcutaneously in nude mice, were significantly increased compared with the control tumors (Fig. 1F). Based on the pan-cancer expression analysis, reduced expression levels of ZNRD1-AS1 were identified in most types of cancer, including LUAD and LUSC (Fig. 1G). Moreover, RT-qPCR demonstrated that ZNRD1-AS1 expression levels in S10, S20 and S30 cells exposed to CS, were significantly downregulated compared with the unexposed BEAS-2B cells in an exposure time-dependent manner (Fig. 1H). The results of the RNA stability assay demonstrated that the degradation rate of ZNRD1-AS1 was decreased in S30 and H1299 cells overexpressing YTHDC2 compared with the control (Blank), whereas it was accelerated following YTHDC2 knockdown (Fig. 1I and J).

Furthermore, ZNRD1-AS1 expression levels was demonstrated to be associated with tumor stage and lymph node metastasis based on the TCGA lung cancer dataset (Figure S2A-C). The association between ZNRD1-AS1 and immune cell infiltration was assessed based on the TCGA lung cancer datasets and XCELL algorithm. The correlation results demonstrated that there was a significant positive correlation between ZNRD1-AS1 expression and CD4 + central memory T cells, mast cells and eosinophils; however, there was a significant negative correlation with lymphoid progenitors, CD4 + T cells, macrophages and monocytes, in both lung cancer subtypes (Figure S2D). Immune checkpoint correlation analysis indicated that ZNRD1-AS1 expression levels were significantly positively correlated with vascular endothelial growth factor A, IL-12A, TNF receptor superfamily member 14 and butyrophilin subfamily 3 member A1, but negatively correlated with CD276, toll-like receptor 4, IL-1A, TNF superfamily member 9, IL-1B and hepatitis A virus cellular receptor 2 (Figure S2E). The survival analysis results demonstrated that among the six probes associated with ZNRD1-AS1, five indicated that patients with high ZNRD1-AS1 expression levels had a better prognosis (HR < 1, Figure S2F).

### YTHDC2 regulates ZNRD1-AS1 via m^6^A modification

**Fig. 2:**
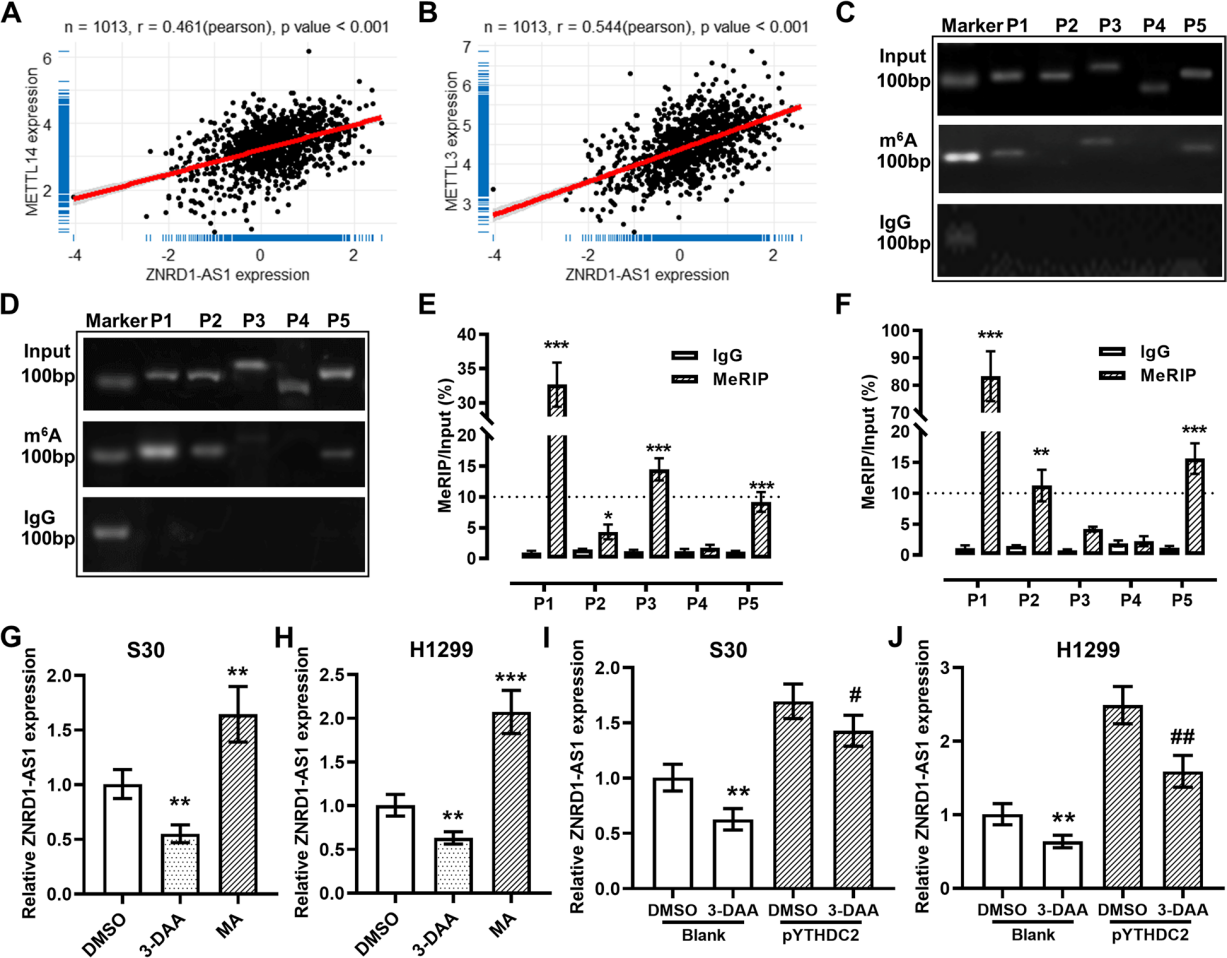
YTHDC2 regulates ZNRD1-AS1 via m^6^A modification. Correlation analysis between the expression of ZNRD1-AS1 and (**A**) METTL14 and (**B**) METTL3 in lung cancer datasets in TCGA database. Five sites with more than moderate confidence were verified by (**C**-**D**) meRIP-PCR and (**E**–**F**) meRIP qPCR in S30 and H1299 cells, the anti-IgG antibody was used as control. * *P* < 0.05, ** *P* < 0.01 and *** *P* < 0.001 vs IgG control group. qPCR was performed to examine the relative expression levels of ZNRD1-AS1 in (**G**) S30 and (**H**) H1299 cells treated with 3-DAA (a global methylation inhibitor) or MA (an FTO inhibitor), for which GAPDH was used as an internal reference. ** *P* < 0.01 and *** *P* < 0.001 vs. DMSO group. qPCR was performed to detect the relative expression levels of ZNRD1-AS1 in 3-DAA-treated YTHDC2 overexpressing (**I**) S30 and (**J**) H1299 cells, ** *P* < 0.01 vs. DMSO blank group, # *P* < 0.05 and ## *P* < 0.01 vs. DMSO pYTHDC2 group. TCGA: The Cancer Genome Atlas. qPCR: quantitative PCR. meRIP: methylated RNA immunoprecipitation

Based on TCGA pan-cancer correlation analysis, it was demonstrated that ZNRD1-AS1 expression was significantly and positively correlated with METTL3 in all tumor types, with the exception of colon adenocarcinoma (Figure S1B), and with METTL14 in all cancer types (R > 0.3, Figure S1C), including LUAD and LUSC (Fig. 2A-B). Using the SRAMP online tool, five predicted m^6^A methylated sites with more than moderate confidence were identified along the ZNRD1-AS1 sequence (Figure S1D). MeRIP-PCR and RT-qPCR results demonstrated that the P1 and P3 sites were significantly enriched in S30 cells (Fig. 2C and E) and P1 and P5 sites were significantly enriched in H1299 cells (Fig. 2D and F). As these results were under the threshold of a meRIP/input > 10%, this indicated the presence of m^6^A methylation modification. Subsequently, the m^6^A level was reduced via 3-DAA treatment and the relative expression level of ZNRD1-AS1 also demonstrated a corresponding significant decrease. Moreover, the relative expression levels of ZNRD1-AS1 were significantly upregulated by MA treatment (Fig. 2G and H). Furthermore, the ZNRD1-AS1 expression upregulation triggered by YTHDC2 was significantly reduced via 3-DAA inhibition, which indicated that YTHDC2 could potentially regulate the stability of ZNRD1-AS1 via m^6^A modification (Fig. 2I and J).

### ZNRD1-AS1 overexpression inhibits cell proliferation, migration and angiogenesis

**Fig. 3:**
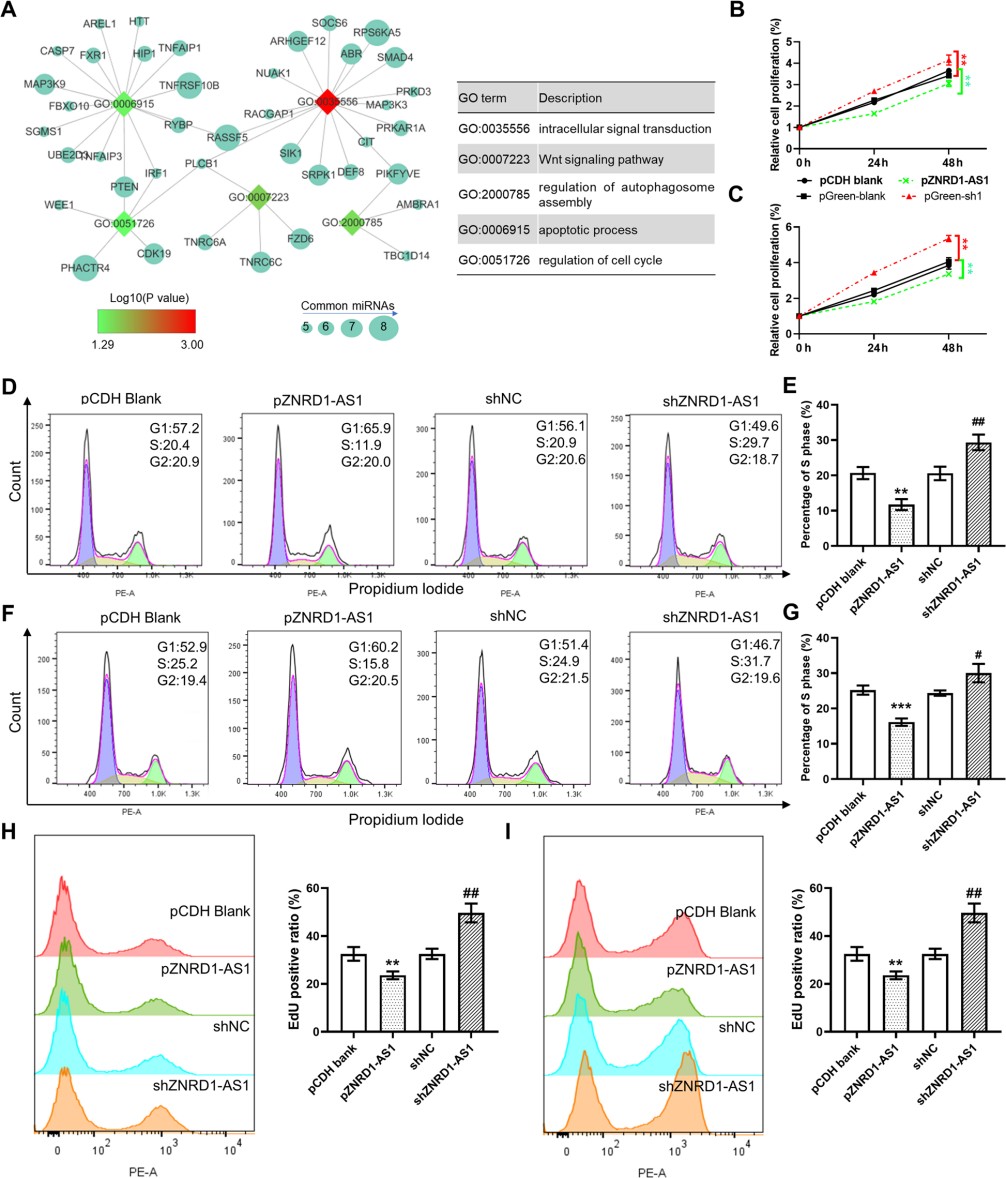
ZNRD1-AS1 knockdown promotes lung cancer cell proliferation. **A** Enrichment analysis of the predicted ceRNAs of ZNRD1-AS1 based on the StarBase online tool. CCK-8 experiments analyzed the proliferation ability of cells following ZNRD-AS1 overexpression or knockdown in (**B**) S30 cells and (**C**) H1299 cells. ** *P* < 0.01 vs. pCDH blank or shNC. Representative images and quantitative results of the cell cycle following knockdown or overexpression of YTHDC2 in (**D** and **E**) S30 cells and (**F**-**G**) H1299 cells. The EdU cell proliferation assay was used to determine cell proliferation ability following knockdown or overexpression of YTHDC2 in (**H**) S30 and (**I**) H1299 cells. ** *P* < 0.01 and *** *P* < 0.001 vs. pCDH blank, and # *P* < 0.05 and ## *P* < 0.01 vs. shNC. shNC: the blank pGreen vector for knockdown

Competing endogenous RNAs (ceRNAs) of ZNRD1-AS1 were predicted using the StarBase online tool. Enrichment analysis demonstrated that these ceRNAs were significantly enriched in several vital biological processes, including the Wnt signaling pathway, autophagosome regulation, apoptosis progression and cell cycle regulation (Fig. 3A). To observe the effect of ZNRD1-AS1 on the proliferation ability of lung tumor cells, the overexpression plasmid (pZNRD1-AS1) and knockdown plasmid of ZNRD1-AS1 (shZNRD1-AS1-1/2) were produced. Overexpression and knockdown efficiencies were verified via qPCR following the transfection of S30 and H1299 cells. The results demonstrated that ZNRD1-AS1 expression levels were significantly upregulated in cells transfected with the overexpression plasmid but were reduced in cells transfected with the knockdown plasmid, compared with the control cells. The results of the CCK-8 assay demonstrated that ZNRD1-AS1 overexpression significantly reduced the proliferation ability of S30 and H1299 cells, whereas knockdown significantly promoted the proliferation ability of both cell lines (Fig. 3B and C). Flow cytometry analysis demonstrated that the proportion of cells in the phase S was significantly decreased following ZNRD1-AS1 overexpression but increased following ZNRD1-AS1 knockdown (Fig. 3D-G). These results were confirmed via the EdU cell proliferation assay, in which ZNRD1-AS1 overexpression significantly decreased the proportion of EdU positive cells, whereas knockdown significantly increased the proportion (Fig. 3H and I).

**Fig. 4:**
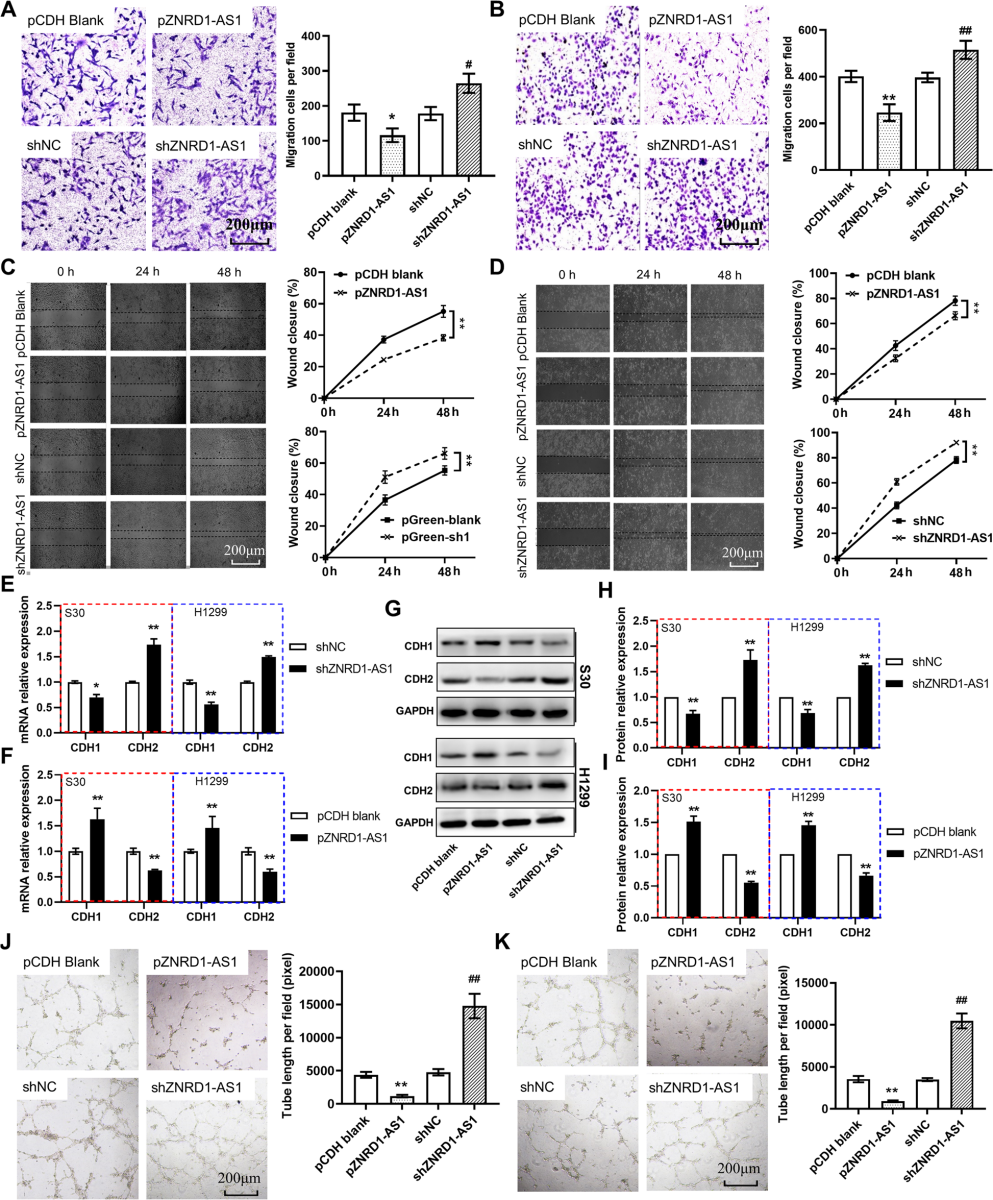
YTHDC2 downregulation promotes lung cancer cell migration and angiogenesis. Representative images and quantitative results of the Transwell migration assay for (**A**) S30 and (**B**) H1299 cells with knockdown or overexpression of ZNRD1-AS1. **C** Representative images and quantitative results for the wound healing assay for (**C**) S30 and (**D**) H1299 cells. RT-qPCR analysis of CDH1 and CDH2 mRNA expression levels in ZNRD1-AS1 (**E**) overexpressed and (**F**) knockdown S30 and H1299 cells. **G**-**I** Protein expression and quantification results of CDH1 and CDH2 in S30 and H1299 cells were determined using western blotting. * *P* < 0.05 and ** *P* < 0.01 vs. pCDH blank or pGreen blank. The HUVEC tube formation assay analyzed the alteration of proangiogenic ability following the overexpression or knockdown of ZNRD1-AS1 in (**J**) S30 and (**K**) H1299 cells. ## *P* < 0.01 vs. pGreen blank. CDH1: E-cadherin. CDH2: N-cadherin. shNC: the blank pGreen vector for knockdown

The cell migration ability was assessed using the Transwell (Fig. 4A and B) and wound-healing assays (Fig. 4C-D). The results demonstrated that a significantly enhanced migration ability was demonstrated in ZNRD1-AS1-knockdown cells. The RT-qPCR and western blotting results demonstrated significantly elevated mRNA and protein expression levels of the epithelial marker CDH1, whereas the expression levels of mesenchymal marker CDH2 were significantly reduced in ZNRD1-AS1-overexpressing cells, compared with the pCDH blank group (Fig. 4E). Moreover, CDH1 expression levels were significantly decreased and CDH2 expression levels were significantly increased in ZNRD1-AS1 knockdown cells compared with the sh-negative control (NC, shNC) group (Fig. 4F). Furthermore, the CDH1 and CDH2 protein expression levels were also showing the consistent change with mRNA level in both ZNRD-AS1 knockdown and overexpressing cells (Fig. 4G-I).

For the in vitro tube formation assay, the medium supernatant of S30 and H1299 cells overexpressing ZNRD1-AS1 significantly inhibited HUVEC cell tube formation and the length of tube structures was also significantly reduced. However, the supernatant of cell culture medium in which ZNRD1-AS1 was knocked down significantly promoted tube formation and the length of tubes was also significantly increased (Fig. 4J and K).

These results suggested that ZNRD1-AS1 may be required for cell proliferation, migration, the EMT process and angiogenesis of lung cancer cells.

### ZNRD1-AS1 upregulation suppresses lung cancer cell tumorigenesis in vivo

**Fig. 5:**
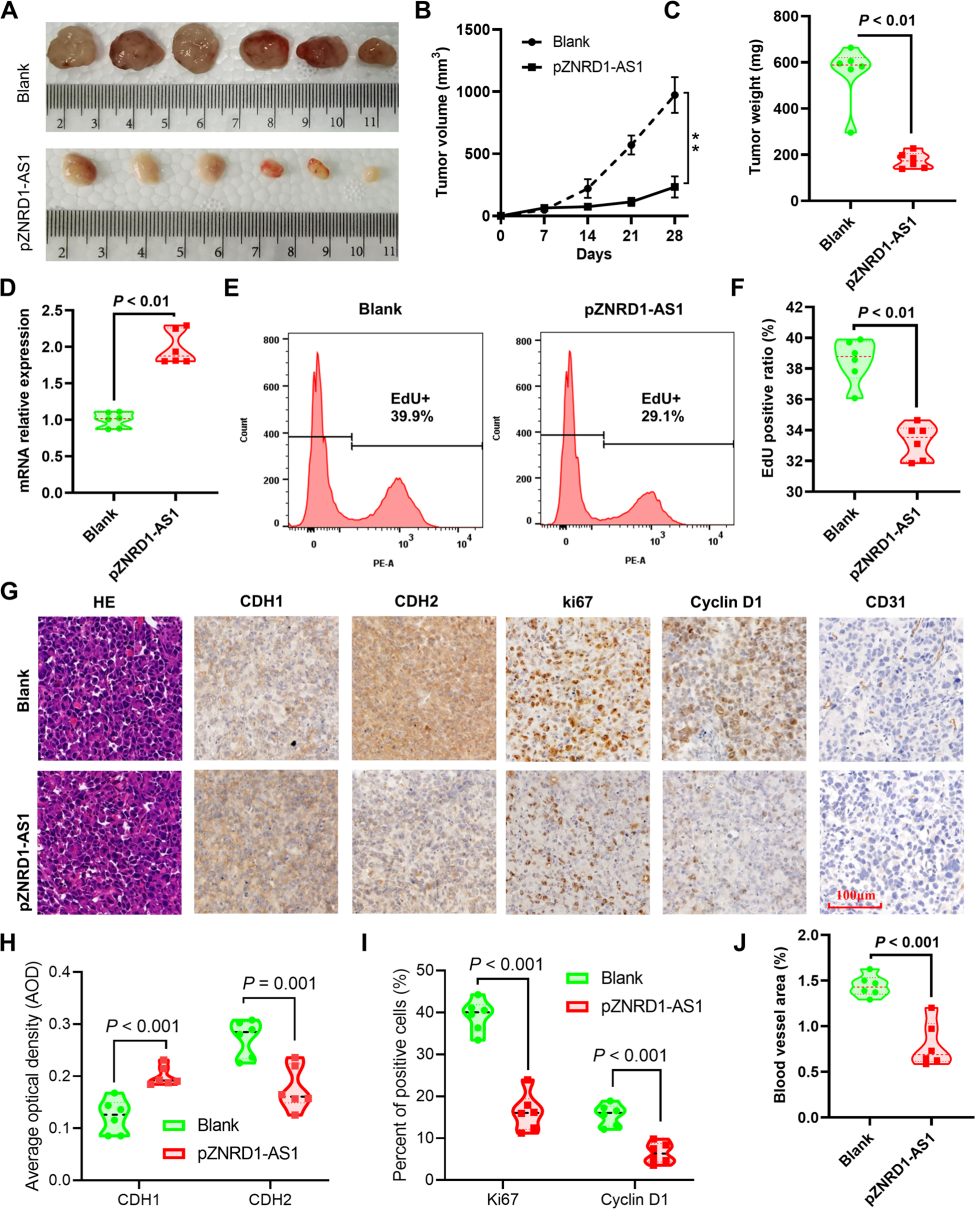
ZNRD1-AS1 overexpression suppresses H1299 cell proliferation in vivo. **A** Images of the xenograft tumors formed in nude mice that were injected with ZNRD1-AS1-overexpressing cells or control cells. The (**B**) volume and (**C**) weight of the xenograft tumors isolated from nude mice. **E** Representative images and the (**F**) quantified result of the EdU cell proliferation assay for single cell suspensions yielded from xenograft tumors. **G** Representative images of H&E staining and the IHC staining of ZNRD1-AS1, Ki-67, cyclin D1, CDH1 and CDH2 in the xenograft tumors derived from nude mice. Scale bar = 100 μm. **H** Average optical density results of CDH1 and CDH2 were quantified using the ImageJ plugin IHC profiler. **I** The percentage of Ki67 and cyclin D1 positive cells was quantified using ImageJ. **J** The blood vessel area of tumor tissue was quantified using ImageJ. **P < 0.01 vs. blank group. IHC: immunohistochemical. CDH1: E-cadherin. CDH2: N-cadherin

The xenograft animal model was used to further investigate the effects of ZNRD1-AS1 on tumor growth in vivo. The results demonstrated that ZNRD1-AS1 overexpression (pZNRD1-AS1) significantly inhibited tumor growth compared with the blank group (Fig. 5A and B). Moreover, the weight of the tumors dissected from the mice in the pZNRD1-AS1 group was significantly lower compared with the blank group (Fig. 5C). The RT-qPCR results validated the elevated ZNRD1-AS1 expression in the pZNRD1-AS1 group (Fig. 5D). The results of the EdU assay demonstrated that there was a significantly lower EdU positive cell ratio in the tumors of the pZNRD1-AS1 group (Fig. 5E-F). Consistent with the in vitro results, the IHC staining of the tumors indicated that CDH1 protein expression levels were increased, whereas CDH2 protein expression levels were decreased in the tumors dissected from the pZNRD1-AS1 mice (Fig. 5G and H). A significantly lower ratio of the Ki67 and cyclin D1 positive cells was found in tumors derived from the pZNRD1-AS1 group (Fig. 5G and I). Furthermore, the neovascular marker CD31 positive area was also found to be markedly reduced in the tumors from the pZNRD1-AS1 group (Fig. 5J).

### miR-942 is a downstream target of ZNRD1-AS1

**Fig. 6:**
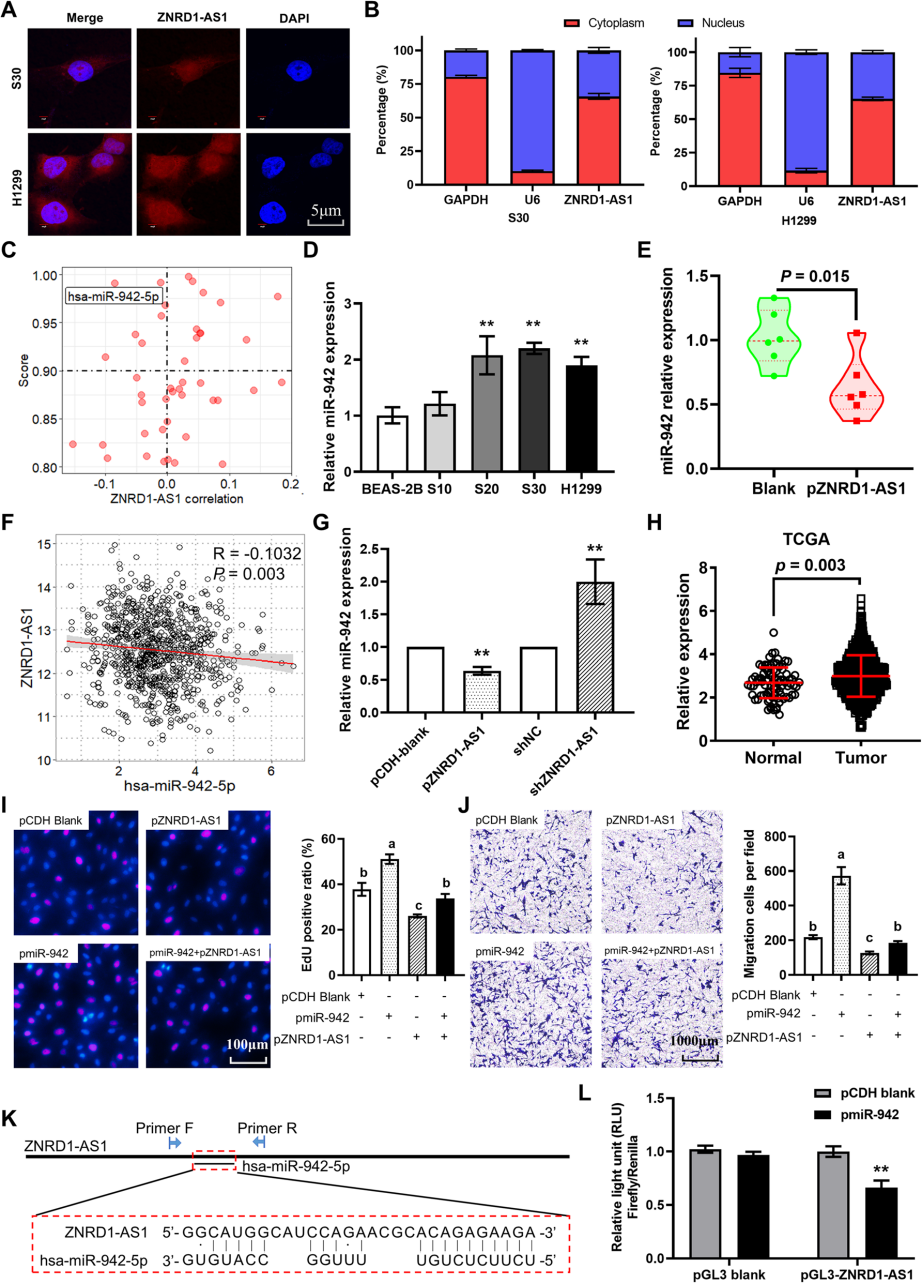
miR-942 is a downstream target of ZNRD1-AS1. **A** FISH was used to analyze the subcellular localization of ZNRD1-AS1 in S30 and H1299 cells. **B** RT-qPCR results of ZNRD1-AS1 expression in the cytoplasm and nucleus of S30 and H1299 cells. **C** Scatter plot showing the screening of ZNRD1-AS1 downstream target miRNAs using the lncBase online tool and correlation analysis. **D** miR-942 expression in CS-exposed cells and H1299 cell. ** *P* < 0.01 vs. BEAS-2B cells. **E** miR-942 expression levels in S30 cells with overexpression or knockdown of ZNRD1-AS1. **F** Correlation analysis of miR-942 and ZNRD1-AS1 in the TCGA lung cancer dataset. ** *P* < 0.01 vs. BEAS-2B group. **G** Expression of miR-942 in subcutaneous tumors formed by ZNRD1-AS1-overexpressing H1299 cells injected into nude mice. ** *P* < 0.01 vs. pCDH blank or pGreen blank. **H** Expression of miR-942 in tumor and normal tissues in TCGA lung cancer dataset. **I** The EdU proliferation assay was performed to analyze the proliferation ability of S30 cells following co-transfection of miR-942 and ZNRD1-AS1 in S30 cells. **J** The Transwell cell migration assay was performed to analyze the altered migration ability of S30 cells following co-transfection of miR-942 and ZNRD1-AS1. Different letters (a, b, c and d) represent statistically significant group differences (*P* < 0.05). **K** The binding sites of ZNRD1-AS1 and miR-942 were predicted using lncBase. (50) The dual-luciferase reporter assay was performed to determine the binding sites of ZNRD1-AS1 and miR-942. TCGA: The Cancer Genome Atlas. CS: cigarette smoke

The results of the FISH assay determined that the hybridization fluorescence signal was observed in both the cytoplasm and nucleus of S30 and H1299 cells (Fig. 6A). Furthermore, RT-qPCR analysis following nucleocytoplasmic fractionation demonstrated that ZNRD1-AS1 was distributed in both the cytoplasm and nucleus, but the amount of ZNRD1-AS1 in the cytoplasm was significantly higher compared with the nucleus (Fig. 6B). To predict the downstream target miRNAs of ZNRD-AS1, the lncBase database was used. Moreover, the association between ZNRD1-AS1 and the identified miRNAs was analyzed based on the lung cancer dataset in the TCGA database. The results demonstrated that miR-942 had a high lncBase score and was significantly negatively correlated with ZNRD1-AS1. mir-942 was therefore identified as the putative target miRNA (Fig. 6C). miR-942 was significantly upregulated in S20, S30 and H1299 cells (Fig. 6D). However, significantly downregulated expression of miR-942 was demonstrated in nude mouse subcutaneous tumors formed by ZNRD1-AS1-overexpressing H1299 cells (Fig. 6E). Furthermore, correlation analysis indicated that ZNRD1-AS1 and miR-942 were significantly negatively correlated in the TCGA lung cancer datasets (Fig. 6F). miR-942 demonstrated a significant downregulation and upregulation following ZNRD-AS1 overexpression or knockdown in S30 cells, respectively (Fig. 6G). Moreover, the analysis of the TCGA lung cancer datasets (including LUAD and LUSC) demonstrated that miR-942 expression levels in lung cancer tissues were significantly higher compare with normal tissues (Fig. 6H). Further analysis revealed that miR-942 expression was significantly correlated with several types of immune cell infiltration (Figure S3A), including stroma score, CD4 T helper 1 cell (Figure S3B), CD4 T helper 2 cell (Figure S3C), CD8 T cell (Figure S3D) and cancer associated fibroblast (Figure S3E).

Rescue experiments were performed to determine the effect of miR-942 on ZNRD1-AS1 function. miR-942 overexpression alone significantly increased the cellular EdU positive ratio and reduced the EdU positive ratio due to the overexpression of ZNRD1-AS1 (Fig. 6I). The results of the Transwell cell migration assay demonstrated that miR-942 overexpression rescued the decrease in cell migration owing to overexpression of ZNRD1-AS1 (Fig. 6J). Subsequently, the putative binding sites of miR-942 to ZNRD1-AS1 were predicted using lncBase (Fig. 6K). Furthermore, the dual-luciferase reporter assay demonstrated that the overexpression of miR-942 significantly reduced the relative fluorescence intensity (Fig. 6L). These results indicated that miR-942 may be a downstream target miRNA of ZNRD1-AS1.

### TNS1 is a ceRNA of ZNRD1-AS1

**Fig. 7:**
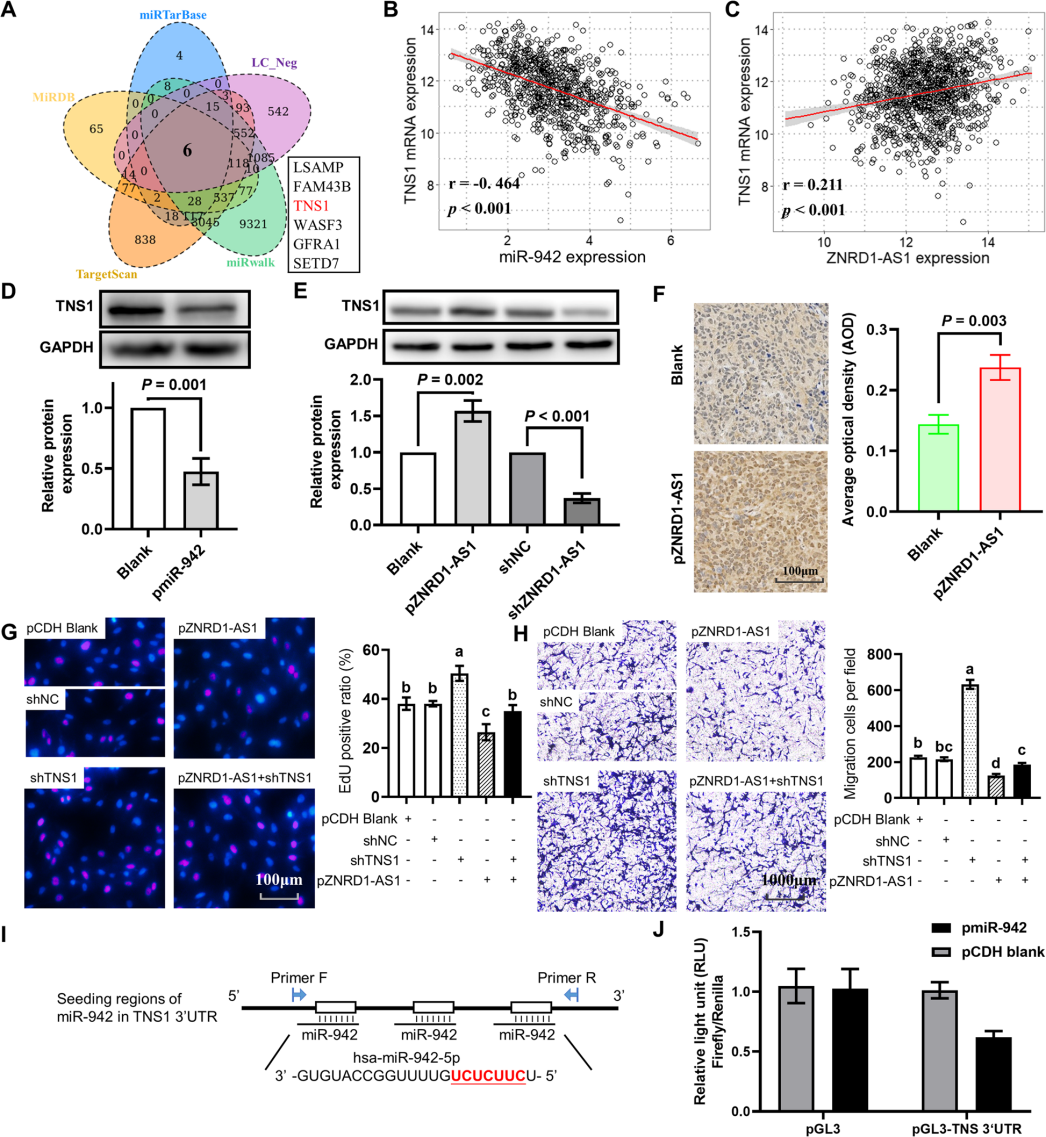
TNS1 is a ceRNA of ZNRD1-AS1. **A** A Venn diagram presents the intersection of miR-942 target genes predicted using four miRNA target gene prediction databases, including miRDB, miRTarBase, TargetScan and miRWalk, as well as the mRNAs correlated with miR-942 expression. Correlation analysis indicated the association between miR-942 and (**B**) TNS1 or (**C**) ZNRD1-AS1 in TCGA lung cancer datasets. Western blotting was performed to determine TNS1 protein expression levels in (**D**) miR-942 overexpressed S30 cells and (**E**) ZNRD1-AS1 overexpressed or knocked-down S30 cells. **F** IHC analysis of TNS1 protein expression levels in subcutaneous tumors formed from ZNRD1-AS1-overexpressed H1299 cells injected into nude mice. **G** The EdU proliferation assay was performed to analyze the proliferation ability of S30 cells following co-transfection of shTNS1 and pZNRD1-AS1 in S30 cells. **H** Transwell cell migration assay analysis of the altered migration ability after co-transfection of shTNS1 and pZNRD1-AS1 in S30 cells. The different letters (a, b, c and d) represent statistically significant group differences (*P* < 0.05). **I** The binding sites between the TNS1 3’UTR and miR-942 were predicted using the TargetScan online tool. **J** The dual-luciferase reporter assay was performed to determine the interaction between TNS1 and miR-942. ceRNAs: Competing endogenous RNAs. IHC: immunohistochemical. UTR: untranslated region

Based on the predicted miR-942 target genes from the four miRNA target prediction databases miRDB, miRTarBase, TargetScan and miRWalk, as well as miR-942 expression-related mRNAs, a total of six intersecting genes were identified. These included the tumor suppressor TNS1 (Fig. 7A). Correlation analysis determined that TNS1 was significantly negatively correlated with miR-942 expression levels (R = -0.4646, Fig. 7B), whereas it was significantly positively correlated with ZNRD1-AS1 expression levels (R = 0.211, Fig. 7C). Moreover, the western blotting results demonstrated that TNS1 expression levels were significantly downregulated in miR-942-overexpressing cells (Fig. 7D). However, ZNRD1-AS1 overexpression was able to significantly upregulate TNS1 protein expression levels, which were also demonstrated to be significantly downregulated following knockdown (Fig. 7E). The upregulation of TNS1 protein expression levels was also observed in nude mouse subcutaneous tumors formed by ZNRD1-AS1-overexpressing H1299 cells (Fig. 7F).

TNS1 expression levels were examined in CS-exposed cells and H1299 cells. The results demonstrated that both TNS1 mRNA and protein expression levels were significantly downregulated in these cells (Figure S4A and B). The downregulation of TNS1 was also demonstrated in two GEO lung cancer datasets, GSE32665 and GSE19188 (Figure S4C and D). Based on the GEPIA online tool, TNS1 was indicated to be significantly downregulated in both lung cancer subtypes compared with normal tissues (Figure S4E). Survival analysis also demonstrated that in patients with lung cancer, high TNS1 expression levels potentially indicated a better prognosis (HR = 0.58, Figure S4F).

Furthermore, rescue assays were used to determine the effect of TNS1 on ZNRD1-AS1 function. TNS1 knockdown significantly increased the cellular EdU positive ratio and rescued the decrease in the EdU positive ratio due to overexpression of ZNRD1-AS1 (Fig. 7G). Transwell cell migration assay also indicated that TNS1 knockdown was able to rescue the decrease in cell migration due to the overexpression of ZNRD1-AS1 (Fig. 7H). The putative binding sites between miR-942 and ZNRD1-AS1 were predicted using the TargetScan online tool (Fig. 7I). The dual-luciferase reporter assay demonstrated that miR-942 overexpression significantly reduced the relative fluorescence (Fig. 7J). These results indicated that miR-942/TNS1 was a downstream target of ZNRD1-AS1.

## Discussion

Increasing evidence has demonstrated that the dysregulation of lncRNAs contributes to enhanced proliferation, metastasis and drug resistance in lung cancer [9, 39, 40]. The aim of the present study was to investigate the biological function and the up- and downstream regulatory mechanisms of ZNRD1-AS1 in lung cancer. A previous study reported that ZNRD1 expression was increased in hepatocellular carcinoma and that ZNRD1 knockdown inhibited cell proliferation, migration and invasion in vitro, as well as tumor growth in vivo [41]. However, the number of studies involving ZNRD1-AS1 is limited and ZNRD1 has more frequently been described as a tumor suppressor gene and has been determined to be highly expressed in tumors [11–13]. For example, in cervical and endometrial cancer, ZNRD1-AS1 can serve a pro-proliferative and migratory role via the downregulation of ZNRD1 [12, 13]. However, in this study the pan-cancer analysis based on TCGA and GTEx databases indicated that ZNRD1-AS1 was significantly downregulated in multiple types of tumor, including LUAD and LUSC. Moreover, clinicopathological analysis indicated that the low expression levels of ZNRD1-AS1 were correlated with lung cancer stage and lymph node metastasis. Furthermore, the results of the present study revealed that ZNRD1-AS1 was also significantly downregulated in CS-exposed cells and H1299 cells. These results were consistent with the aforementioned studies, whereby ZNRD1-AS1 served as a tumor suppressor gene. Moreover, survival analysis based on a large sample in the KMPlotter online tool also demonstrated that patients with high ZNRD1-AS1 expression levels lung cancer exhibited an improved prognosis. At each step of carcinogenesis, cancer cells are exposed to the immune system, which attacks them to inhibit their proliferation [42]. Therefore immune infiltration and immune checkpoint analysis were performed. These results indicated that there was a correlation between ZNRD1-AS1 and tumor immunity. These data suggested that ZNRD1-AS1 may be of great clinical significance in lung cancer.

lncRNA function is often associated with their subcellular localization [43]. In the present study ZNRD1-AS1 was shown to be distributed both in the cytoplasm and nucleus, but mainly in the cytoplasm. This distribution therefore provided a basis for determining its downstream ceRNA regulatory mechanism. Bioinformatics analysis, as well as further cellular experiments, confirmed that ZNRD1-AS1 was able to significantly inhibit cell proliferation and migration. A recent study reported that ZNRD1-AS1 has a ceRNA mechanism to regulate angiogenesis in glioma [44]. In the present study, the overexpression of ZNRD1-AS1 was demonstrated to inhibit tube formation ability, which was determined via the in vitro HUVEC cell tube formation assay and IHC analysis of the neovascular marker CD31. Furthermore, subcutaneous tumorigenesis experiments in nude mice further confirmed the tumor growth inhibitory effect of ZNRD1-AS1. ZNRD1-AS1 also functions in nasopharyngeal carcinoma to accelerate cell metastasis and invasion via a ceRNA mechanism, by which miR-335 regulates rho-associated protein kinase-1 expression [45]. Under the conditions of the present study, ZNRD1-AS1 was demonstrated to target miR-942 and thus regulate the expression of its ceRNA TNS1, which thereby modulated the proliferation and migration abilities of malignant lung cells. miR-942 has been reported to regulate the development of a variety of tumors, serving a role in promoting tumor cell proliferation and migration in numerous types of tumor [46–48]. Several studies have also reported that the loss of TNS1 can lead to a reduction in the migratory ability of numerous types of cancer cells [49, 50]. Furthermore, TNS1 has also been demonstrated to be associated with the EMT process of cancer cells [51]. The results of the present study demonstrated that ZNRD1-AS1 can regulate the proliferation and migration abilities of malignant lung cells via the miR-942/TNS1 axis.

Previous studies have reported that m^6^A methylation can affect numerous tumor cell properties via the regulation of lncRNAs. In liver cancer, enhancing LINC00958 stability via METTL3 through m^6^A modification leads to its upregulation, thereby promoting cancer progression [24]. The demethylase ALKBH5 can also regulate the expression of KCNK15-AS1 via demethylation, which inhibits pancreatic cancer cell migration and invasion [25]. In ovarian cancer, YTH domain-containing family protein 1 (YTHDF1) promotes the translation of eukaryotic translation initiation factor 3 subunit C to promote tumorigenesis and metastasis [52]. However, in melanoma YTHDF1 may act as a tumor suppressor that can promote the translation of the tumor suppressor gene histidine triad nucleotide binding protein 2 to inhibit tumor development [53]. For YTHDC2, its knockdown attenuates soft agar clonogenic and metastatic abilities of breast cancer cells [54]. Our previous study revealed that YTHDC2 is a cigarette smoking-drived gene in lung cancer, and it functions as a tumor suppressor through the CYLD/NF-κB signaling pathway, which is mediated by m^6^A modification [26]. The present study demonstrated that m^6^A modification occurs on ZNRD1-AS1 and YTHDC2 potentially binds to ZNRD1-AS1 and promotes its stability. Interference with m^6^A modification via treatment with the m^6^A inhibitor 3-DAA and demethylase FTO inhibitor MA regulated the abundance of ZNRD1-AS1 in cells. These results demonstrated that ZNRD1-AS1 is potentially regulated via m^6^A modification and YTHDC2.

In summary, the present study illustrated the effects of ZNRD1-AS1 on lung cancer cell proliferation and migration and demonstrated its clinical significance via in vitro and in vivo experiments and bioinformatics analysis. Furthermore, its upstream regulatory mechanism was further explored from an epigenomic perspective and the results demonstrated that it harbored m^6^A modification sites and was regulated by the ‘reader’ protein YTHDC2 under m^6^A mediation. A better understanding of m^6^A modification of ZNRD1-AS1 will provide more insight into the underlying molecular mechanisms of lung cancer and facilitate the development of more effective personalized treatment strategies.

## Supplementary Information

**Additional file 1:** **Table S1. **Primer pairs designedfor ZNRD1-AS1 segmental amplification. **Figure S1.** ZNRD1-AS1 ispredicted to be regulated via m^6^A modification. Scatter plots presentingthe correlation analysis between ZNRD1-AS1 and the m^6^A ‘reader’ (A)YTHDC2 as well as m^6^A ‘writers’ (B) METTL3 and (C) METTL14 viapan-cancer analysis. (D) m^6^A sites in ZNRD1-AS1, which werepredicted using the SRAMP online tool. **Figure S2.** Clinicalsignificance of ZNRD1-AS1 in lung cancer. Relative expression levels ofZNRD1-AS1 in LUAD tissues at (A) different pathological stages and in (B) lymphnode metastasis based on TCGA database. (C) Relative expression levels ofZNRD1-AS1 in LUSC tissues with different lymph node metastasis based on theTCGA database. (D) Spearman correlation analysis between ZNRD1-AS1 and immunecell infiltration levels based on the XCELL algorithm in LUAD and LUSCdatasets. (E) Spearman correlation analysis between the expression levels ofZNRD1-AS1 and immune checkpoint genes in LUAD and LUSC datasets. (F) Probabilityof overall survival in patients with lung cancer expressing high or lowZNRD1-AS1 levels according to KMplotter online tool. **Figure S3.** miR-942 associationwith immune cell infiltration in lung cancer. (A) Scatter plot presenting thecorrelation analysis between miR-942 expression and immune cell infiltrationscore using the XCELL algorithm in LUSC and LUAD datasets. Scatter plots presentingthe association between miR-942 expression levels and (B) CD4+ T helper 1 cells,(C) CD4+ T helper 2 cells and (D) T cell CD8+ naïve cells, as well as (E) cancerassociated fibroblasts in lung cancer. **Figure S4.** TNS1 wasdownregulated in CS-exposed cells and lung cancer tissues. Relative TNS1 (A)mRNA and (B) protein expression in CS-exposed cells. ** *P < *0.01 and *** *P < *0.001vs. BEAS-2B cells. TNS1 was demonstrated to be downregulated in lung cancerbased on the (C) GSE32665 and (D) GSE19188 datasets from the GEO database, aswell as in (E) LUAD and LUSC datasets from the TCGA database. *** *P < *0.001vs. normal. (F) Probability ofoverall survival in patients with lung cancer expressing high or low TNS1expression levels using the KMplotter online tool.

## Data Availability

All data generated and described in this article are available from the corresponding web servers, and are freely available to any scientist wishing to use them for noncommercial purposes, without breaching participant confidentiality. All codes and R-packages used in the study are publicly available and have been disclosed in Methods or are available from the corresponding authors on reasonable request.

## Abbreviations

LncRNA: Long non-coding RNA
RIP: RNA immunoprecipitation
LUAD: Lung adenocarcinoma
LUSC: Lung squamous cell carcinoma
qPCR: Quantitative PCR
siNC: Negative control for siRNA transfection
TCGA: The Cancer Genome Atlas
GTEx: The Genotype-Tissue Expression
CS: Cigarette smoke
IHC: Immunohistochemical
EMT: Epithelial-mesenchymal transition
CDH1: E-cadherin
CDH2: N-cadherin
shNC: The blank pGreen vector for knockdown
meRIP: Methylated RNA immunoprecipitation
ceRNAs: Competing endogenous RNAs
UTR: Untranslated region
TPM: Transcripts per million
SPF: Specific pathogen-free
