# Supplementary material for: lncRNA ZNRD1-AS1 promotes malignant lung cell proliferation, migration, and angiogenesis via the miR-942/TNS1 axis and is positively regulated by the m6A reader YTHDC2
Source: Mol Cancer. 2022 Dec 30;21:229. doi: 10.1186/s12943-022-01705-7 (PMC9801573; doi:10.1186/s12943-022-01705-7)
Supplement: Supplementary file 1 — Additional file 1: Table S1. Primer pairs designedfor ZNRD1-AS1 segmental amplification. Figure S1. ZNRD1-AS1 ispredicted to be regulated via m6A modification. Scatter plots presentingthe correlation analysis between ZNRD1-AS1 and the m6A ‘reader’ (A)YTHDC2 as well as m6A ‘writers’ (B) METTL3 and (C) METTL14 viapan-cancer analysis. (D) m6A sites in ZNRD1-AS1, which werepredicted using the SRAMP online tool. Figure S2. Clinicalsignificance of ZNRD1-AS1 in lung cancer. Relative expression levels ofZNRD1-AS1 in LUAD tissues at (A) different pathological stages and in (B) lymphnode metastasis based on TCGA database. (C) Relative expression levels ofZNRD1-AS1 in LUSC tissues with different lymph node metastasis based on theTCGA database. (D) Spearman correlation analysis between ZNRD1-AS1 and immunecell infiltration levels based on the XCELL algorithm in LUAD and LUSCdatasets. (E) Spearman correlation analysis between the expression levels ofZNRD1-AS1 and immune checkpoint genes in LUAD and LUSC datasets. (F) Probabilityof overall survival in patients with lung cancer expressing high or lowZNRD1-AS1 levels according to KMplotter online tool. Figure S3. miR-942 associationwith immune cell infiltration in lung cancer. (A) Scatter plot presenting thecorrelation analysis between miR-942 expression and immune cell infiltrationscore using the XCELL algorithm in LUSC and LUAD datasets. Scatter plots presentingthe association between miR-942 expression levels and (B) CD4+ T helper 1 cells,(C) CD4+ T helper 2 cells and (D) T cell CD8+ naïve cells, as well as (E) cancerassociated fibroblasts in lung cancer. Figure S4. TNS1 wasdownregulated in CS-exposed cells and lung cancer tissues. Relative TNS1 (A)mRNA and (B) protein expression in CS-exposed cells. ** P < 0.01 and *** P < 0.001vs. BEAS-2B cells. TNS1 was demonstrated to be downregulated in lung cancerbased on the (C) GSE32665 and (D) GSE19188 datasets from the GEO database, aswell as in (E) LUAD and LUSC datasets from the TCGA [file 12943_2022_1705_MOESM1_ESM.docx]

# Supplementary tables and figures

Table S1 Primer pairs designed for ZNRD1-AS1 segmental amplification

| Segment | Primer_F | Primer_R |
| --- | --- | --- |
| Seg1 | CGGAATTCCGCGAACCGTGTCTCGGGAG | CGGGATCCCGCTAAATGGTTTGTTCTTAAATTCTCC |
| Seg2 | CGGAATTCCGACAAGTACTACAACCCCCCA | CGGGATCCCGAGAACGAGCATTTGAAAAAAAAAAGG |
| Seg3 | CGGAATTCCGTCCAGGGTTCAGTGAATA | CGGGATCCCGTATTCACCTTCAGTAACTAACAT |
| Seg4 | CGGAATTCCGAAGAGAAGTCTCTGCCCTTG | GCTCTAGAGCCAACTTAAGCATAATATTCCTGAGGG |
| Seg5 | CGGAATTCCGTGATAATTCTGATGCCCACC | CGGGATCCCGAAATTCCAGAACTAGCAAAGAG |
| Seg6 | CGGAATTCCGCCAGGATGCACATCAAAA | CGGGATCCCGTAAGCAACTGTTAGGGATAC |


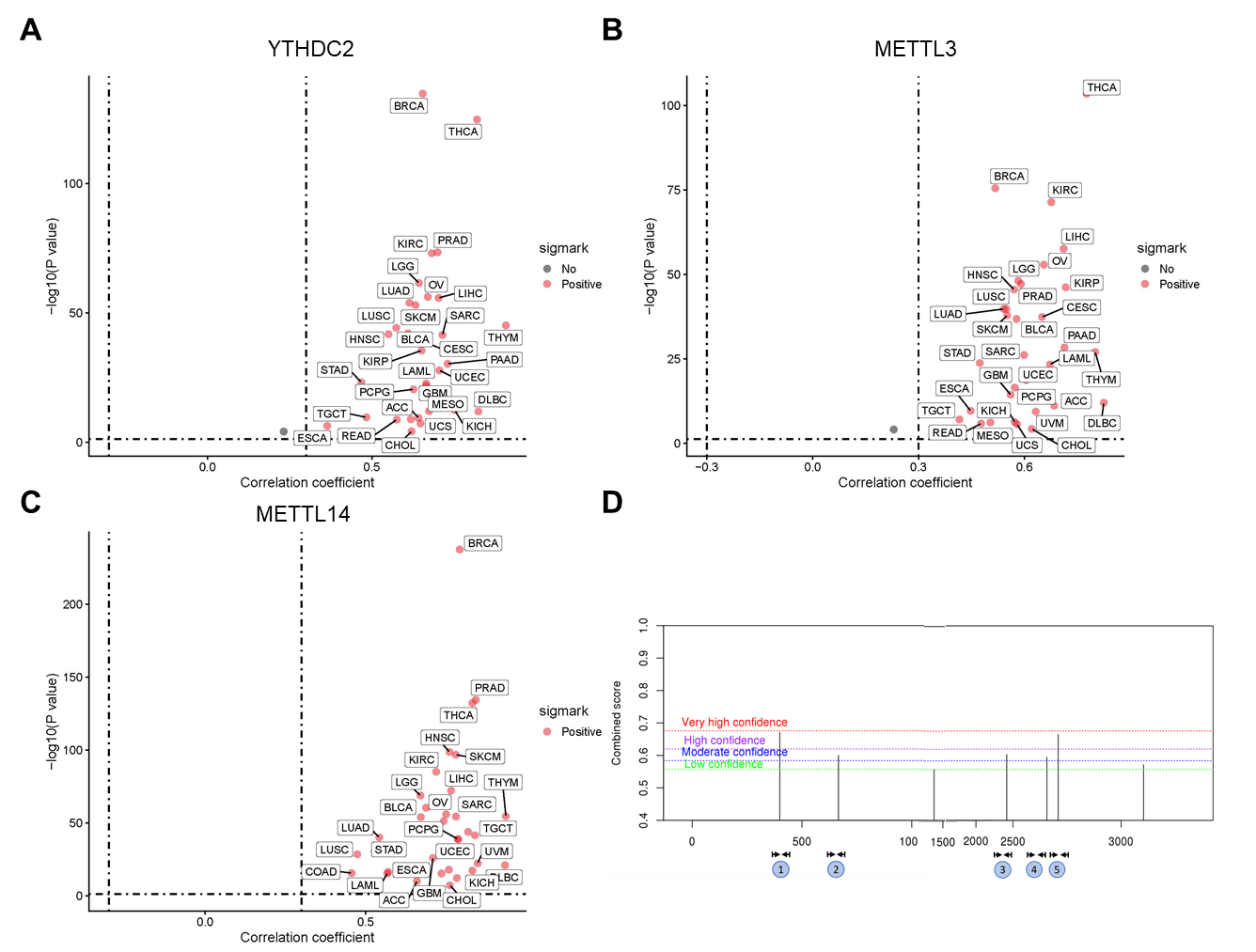


**Figure S1** ZNRD1-AS1 is predicted to be regulated via m^6^A modification. Scatter plots presenting the correlation analysis between ZNRD1-AS1 and the m^6^A ‘reader’ (A) YTHDC2 as well as m^6^A ‘writers’ (B) METTL3 and (C) METTL14 via pan-cancer analysis. (D) m^6^A sites in ZNRD1-AS1, which were predicted using the SRAMP online tool.


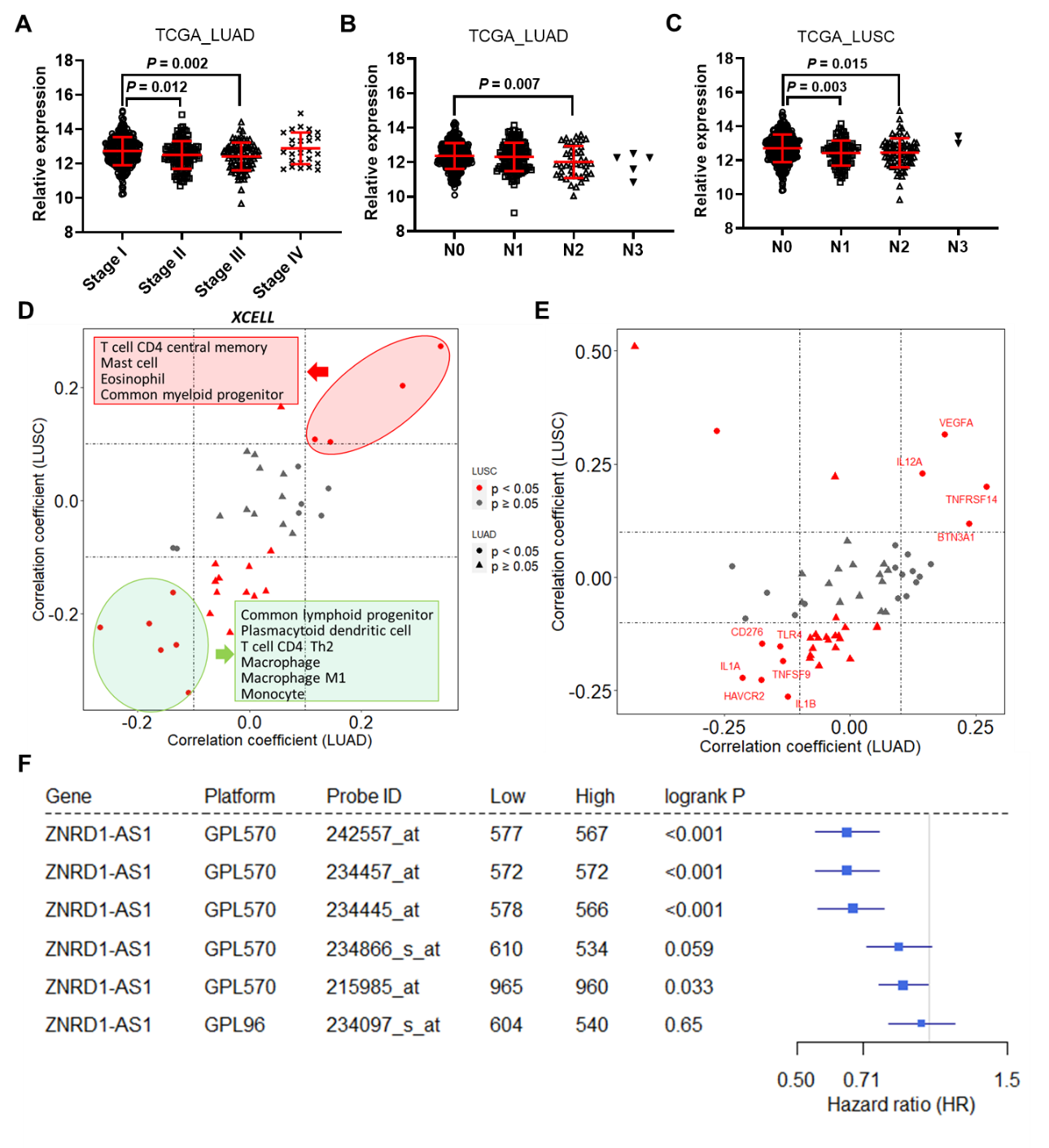


**Figure S2** Clinical significance of ZNRD1-AS1 in lung cancer. Relative expression levels of ZNRD1-AS1 in LUAD tissues at (A) different pathological stages and in (B) lymph node metastasis based on TCGA database. (C) Relative expression levels of ZNRD1-AS1 in LUSC tissues with different lymph node metastasis based on the TCGA database. (D) Spearman correlation analysis between ZNRD1-AS1 and immune cell infiltration levels based on the XCELL algorithm in LUAD and LUSC datasets. (E) Spearman correlation analysis between the expression levels of ZNRD1-AS1 and immune checkpoint genes in LUAD and LUSC datasets. (F) Probability of overall survival in patients with lung cancer expressing high or low ZNRD1-AS1 levels according to KMplotter online tool.


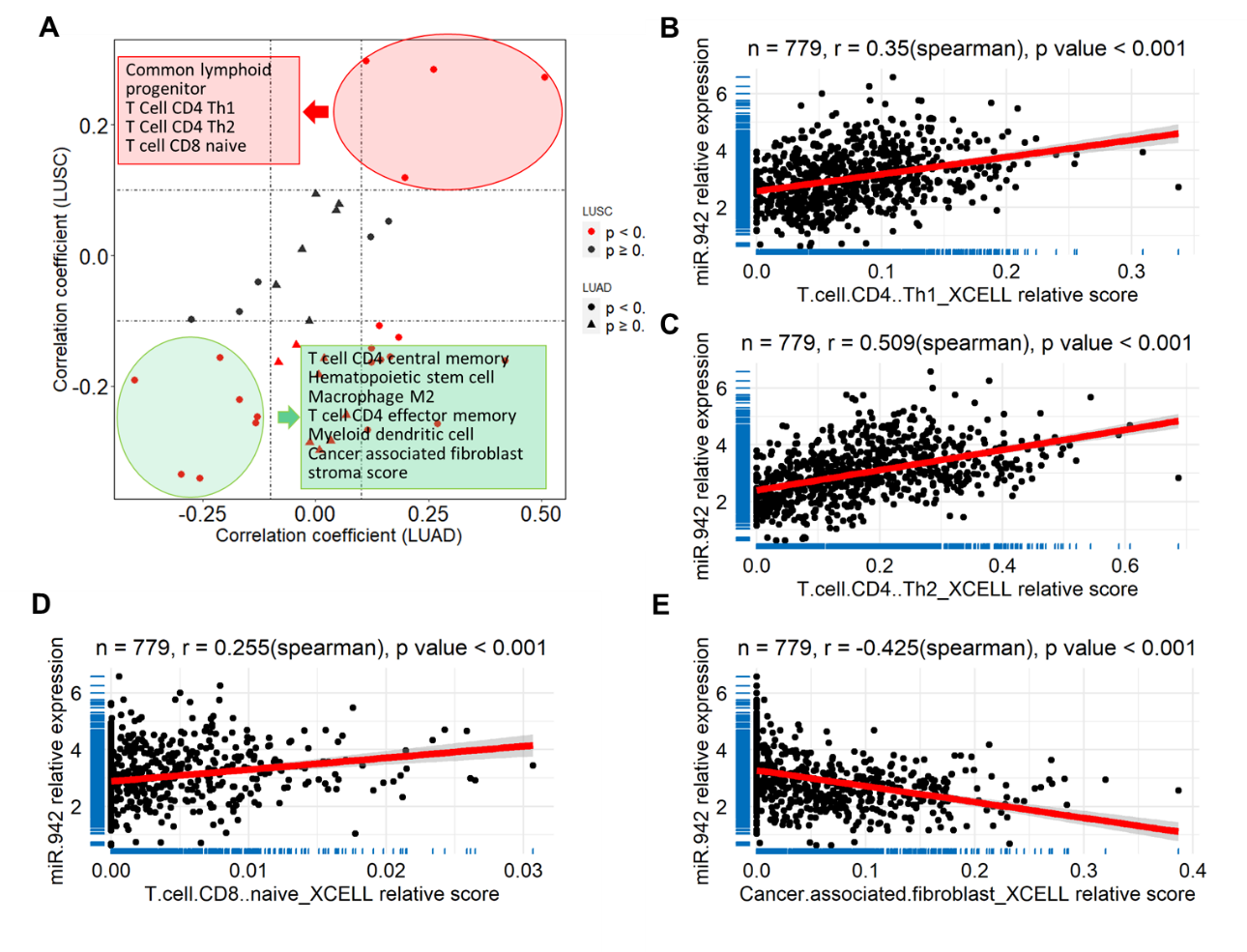


**Figure S3** miR-942 association with immune cell infiltration in lung cancer. (A) Scatter plot presenting the correlation analysis between miR-942 expression and immune cell infiltration score using the XCELL algorithm in LUSC and LUAD datasets. Scatter plots presenting the association between miR-942 expression levels and (B) CD4+ T helper 1 cells, (C) CD4+ T helper 2 cells and (D) T cell CD8+ naïve cells, as well as (E) cancer associated fibroblasts in lung cancer.


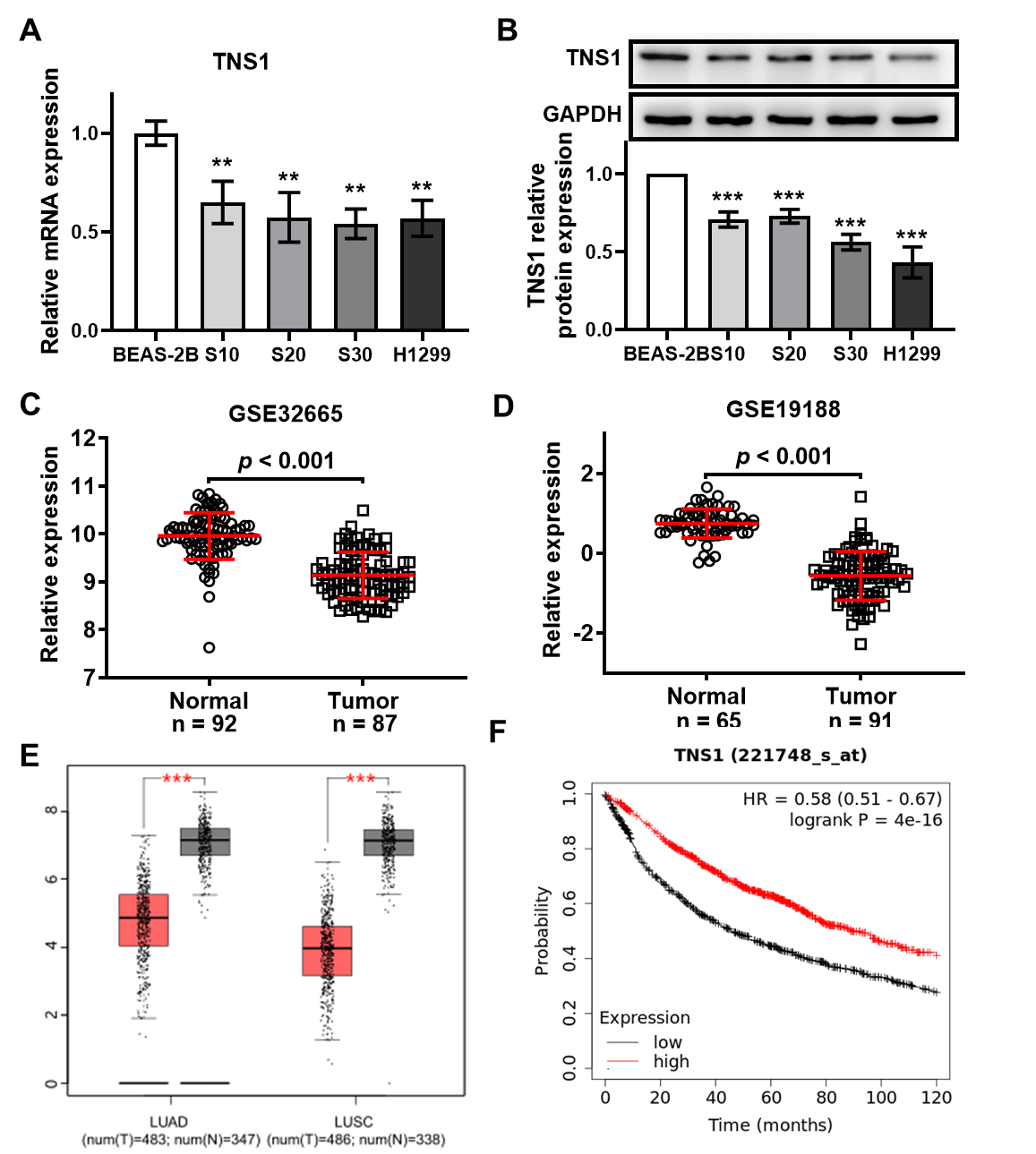


**Figure S4** TNS1 was downregulated in CS-exposed cells and lung cancer tissues. Relative TNS1 (A) mRNA and (B) protein expression in CS-exposed cells. ** *P <* 0.01 and *** *P <* 0.001 vs. BEAS-2B cells. TNS1 was demonstrated to be downregulated in lung cancer based on the (C) GSE32665 and (D) GSE19188 datasets from the GEO database, as well as in (E) LUAD and LUSC datasets from the TCGA database. *** *P <* 0.001 vs. normal. (F) Probability of overall survival in patients with lung cancer expressing high or low TNS1 expression levels using the KMplotter online tool.
